# *De novo* transcriptome assembly associated with fumonisin production by the rice pathogen *Fusarium fujikuroi*

**DOI:** 10.1016/j.dib.2018.02.072

**Published:** 2018-03-06

**Authors:** Keerthi S. Guruge, Ryuichi Uegaki

**Affiliations:** a Pathology and Pathophysiology Research Division, National Institute of Animal Health, National Agriculture and Food Research Organization, Kannondai 3-1-5, Tsukuba 305-0856, Japan; b Graduate School of Life and Environmental Sciences, Osaka Prefecture University, Osaka, Japan; c Centre for Crop Health, University of Southern Queensland, Toowoomba Campus, QLD 4350, Australia

**Keywords:** *Fusarium fujikuroi*, Fumonisin, Next-generation sequencing, Transcriptome, Gene-expression

## Abstract

The present study employed a next-generation sequencing method to assemble a *de novo* transcriptome database designed to distinguish gene expression changes exhibited by the fumonisin-producing fungus *Fusarium fujikuroi* when grown under ‘fumonisin-producing’ compared to ‘non-fumonisin-producing’ conditions. The raw data of this study have been deposited at DNA Data Bank of Japan (DDBJ) under the accession ID DRA006146.

**Specifications Table**

**Table:** 

Subject area	Agriculture, Food safety
More specific subject area	Mycotoxins; Genes induced during fumonisin production
Type of data	Table, text file, figure
How data was acquired	*De novo* transcriptome assembly was constructed using next-generation mRNA sequencing techniques using by the Illumina HiSeq 2000 sequencing system
Data format	Raw (FASTQ) sequences
Experimental factors	A *F. fujikuroi* strain was cultured for 7 days for total RNA extraction, sequencing, *de novo* transcriptome assembly and annotation
Experimental features	The gene expression changes exhibited by the fumonisin-producing fungus *F. fujikuroi* when grown under ‘fumonisin-producing’ compared to ‘non-fumonisin-producing’ conditions were studied
Data source location	Kanto region, Japan
Data accessibility	The raw data have been deposited at DNA Data Bank of Japan (DDBJ). http://trace.ddbj.nig.ac.jp/DRASearch/submission?acc=DRA006146

**Value of the data**

• The novel data base was constructed from a *F. fujikuroi* fungal strain which is postulated to be a source of fumonisin contamination of major crops such as rice and corn produced in Japan, due to its high prevalence in the country.
• The data presents an assemble of a *de novo* transcriptome library, with the aim of identifying changes to the gene expression profile of *F. fujikuroi* specimens grown under “fumonisin-producing” versus “non-fumonisin-producing”’ culture conditions.
• Further analysis of the data presented here may enable the identification of novel genomic networks, and thereby promote a better understanding of the fumonisin induction pathway.

## 1 Data

Data reported here describe the fumonisin concentration, sequencing results obtained from the *de novo Fusarium fujikuroi* mRNA transcriptome (Table 1) and the conducted Gene Ontology (GO) functional analysis (Supplementary Fig. 1a-c). In addition, up- and down-regulated (> 2-fold) genes under the two culture methods on fumonisin production were acquired by homology search using the tblastn (NCBI rice bakanae disease protein database) (Supplementary Table 1). A total of four raw sequence data were deposited into DDBJ DRA data base and can be accessed with the Bio Project accession number PRJDB6333.

**Table 1:** Contig assembly summary for the *de novo F. fujikuroi* mRNA transcriptome.

Total trinity 'genes'	20,122
Total trinity transcripts	26,014
Percent GC	49.92
N90	787
N50	3185
N10	7210
Maximum contig length	22,049
Minimum contig length	201
Average contig length	1699.71
Total assembled bases	44,216,316

(http://trace.ddbj.nig.ac.jp/BPSearch/bioproject?acc=PRJDB6333) under the Bio Sample accession numbers SAMD00093543.

(http://trace.ddbj.nig.ac.jp/BSSearch/biosample?acc=SAMD00093543) and SAMD00093544 (http://trace.ddbj.nig.ac.jp/BSSearch/biosample?acc=SAMD00093544).

### 1.1 Fumonisin (FB1) concentration

No FB1 was detected (< 1 μg/L) either at the initiation of, or after 7 days of MO409 strain exposure to the non-fumonisin-producing growth conditions. In contrast, the concentration of FB1 reached 13 and 940 μg/L at the initiation of, and after 7 days of MO409 strain exposure to the fumonisin-producing growth conditions, respectively.

## 2 Experimental design, materials, and methods

### 2.1 Fungal culture and sampling

The present study cultured the wild-type *F. fujikuroi* strain MO409 [which was previously isolated from a Japanese rice specimen (1)] under established “fumonisin-producing” and “non-fumonisin-producing” culture conditions. The MO409 strain was initially maintained in V-8 mix (Canbel) agar (Wako pure chemical, Osaka, Japan) medium at 25 °C for 7 days. Sterile purified water (5 mL) was then added to this mix, and the culture was scraped well to produce a spore solution whose content was adjusted to 1.25 × 10^8^ CFU. A 50 μL aliquot of this pre-culture solution was then transferred to 50 mL of either non-fumonisin-producing or fumonisin-producing medium, comprised of YM broth only (Difco Laboratories Detroit MI, USA) and YM broth supplemented with 0.5% v/v corn steep liquor (Sigma-Aldrich, St. Louis, MO, USA), respectively. The generated cultures were then incubated at 25 °C for 7 days.

### 2.2 RNA isolation

After 1 week, the culture medium was transferred to a 50 mL PP centrifuge tube, and centrifuged at 3000 rpm for 15 min (Beckman CS-6KR, Tokyo, Japan). The resulting supernatant was removed, and stored at − 20 °C until further analysis (see Section 2.3). The fungal pellet was then cryo-crushed in liquid nitrogen using a mortar and pestle, and immediately transferred to a vial with 0.5 mL of Lysis buffer (QuickGene RNA Tissue Kit S II (RT-S2); Fuji Film, Tokyo, Japan). The total RNA was extracted from each fungal sample using the QuickGene-810 Nuclear Acid Isolation System (Fuji Film) according the to manufacturer's instructions (with DNAse-treatment). The purity of each extracted RNA sample was evaluated by the optical density ratio at 260 and 280 nm, and the total RNA concentration in each sample was determined by measuring its absorbance at 260 nm (BioTek Instrument, Tokyo, Japan). All RNA samples then were stored at − 80 °C for subsequent NGS analysis. Two RNA samples isolated from each of the culture conditions, which were calculated to have an RNA integrity number (RIN) of 10, were used to prepare a cDNA library. All experiments were conducted under sterile conditions.

### 2.3 Fumonisin quantification

The fumonisin (FB1) content of the stored MO409 supernatant (see Section 2.2) was quantified using a high-performance liquid chromatography – tandem mass spectrometry (HPLC-MS/MS) system, as previously described [1]. Briefly, 2 mL of supernatant was mixed with 6 mL of methanol and loaded into anion-exchange column (Bond Elut SAX; Varian Technologies, Palo Alto, CA, USA), preconditioned with 10 mL of water and methanol (1:3 v/v), and eluted with 14 mL of methanol containing 1% (v/v) acetic acid. The eluate was concentrated to 1 mL and injected into the HPLC-MS/MS system (Acquity UPLC; Waters, Milford, MA, USA) equipped with Acquity UPLC BEH C18 column (Waters, Milford, MA, USA).

### 2.4 NGS analysis

*De novo* transcriptome sequencing was performed by Macrogen (Tokyo, Japan). cDNA libraries were prepared from the mRNA samples collected under growth conditions (*i.e*. fumonisin-producing and non-fumonisin-producing) after fumonisin quantification. In brief, the cDNA libraries were separately prepared using the reagents provided in the TruSeq RNA Sample Preparation Kit (Illumina). Initially, poly-A mRNA was purified using the poly-T oligo-attached magnetic beads. Next, the extracted mRNA was fragmented, and transcribed to cDNA using reverse transcriptase and random primers. Second-strand cDNA synthesis was achieved using DNA Polymerase I and RNase H, before the generated cDNA fragments were subjected to an “end-repair” process that comprised the addition of a single “A” base, followed by adapter ligation. The resultant products were then purified, and enriched via PCR to produce a cDNA library. After a quality control analysis was conducted on a sample library, and the DNA library templates were quantified, sequencing was performed using by the Illumina HiSeq. 2000 sequencing system (paired-end mode), which was automated by Sequencing-by-Synthesis technology software (Solexa). The quality of the conducted sequencing was assessed using the FastQC (http://www.bioinformatics.babraham.ac.uk/projects/fastqc) and Trimmomatic (0.32) (http://www.usadellab.org/cms/?page=trimmomatic) software tools. The *de novo* reconstruction of transcriptomes from RNA-seq data was then performed using the Trinity (r20140717) software tool, and likewise, the transcript abundance was quantified from using the RNA-Seq by Expectation-Maximization (RSEM: 1.2.15) software tool. Finally, nucleotide query sequences for both strands were annotated against the NCBI protein sequence database (go_v20150407) using the BlastX (Gene Ontology) software tool.

### 2.5 Transcriptome sequencing, alignment, and mapping

The analyses of *F. fujikuroi* strains maintained under fumonisin-producing and non-fumonisin-producing conditions generated 4,101,500,314 and 5,562,688,302 raw, and 4,071,113,473 (GC%, 52%; Q20%, 98.5) and 5,443,010,225 (GC%, 52%; Q20%, 98.4) trimmed reads, respectively. These were used to select 26,014*F. fujikuroi* transcripts which had a contig length of 201-22,049 bases (N50, 3185 bases), and an average GC content of 49.92% (Table 1).

### 2.6 Gene Ontology (GO) functional analysis

The generated transcripts were next subjected to various pre-processing methods (including data filtering, transformation, and normalization) to preclude statistically erroneous conclusions. Of the 26,014 contigs, 3367 were excluded, whereas 22,647 were selected for further analysis. The retained *de novo* data exhibited similarities with 17,210 annotated genes in the National Centre for Biotechnology Information (NCBI) protein database, (although 5437 of the genes included in the de novo assembly did not share similarity with any NCBI entries). A conducted Gene Ontology (GO) functional analysis of the F. fujikuroi transcripts revealed that 26%, 21%, and 21% of the similarities were found to occur with genes previously shown to be associated with cellular components, molecular functions, and biological processes, respectively (Supplementary Fig. 1a-c).
