# Supplementary material for: De novo transcriptome assembly associated with fumonisin production by the rice pathogen Fusarium fujikuroi
Source: Data Brief. 2018 Mar 6;18:35–9. doi: 10.1016/j.dib.2018.02.072 (PMC5996136; doi:10.1016/j.dib.2018.02.072)
Supplement: Supplementary file 2 — Supplementary material [file mmc2.pdf]

Data in Brief

Electronic Supplementary Material

De novo **transcriptome assembly associated with fumonisin production by the rice pathogen** ***Fusarium fujikuroi***

Keerthi S. Guruge, Ryuichi Uegaki

Supplementary figure 1. Gene ontology (GO) enrichment of global gene expression of *Fusarium fujikuroi*. The results are summarized in three main categories: cellular component (a), biological process (b), molecular function (c).

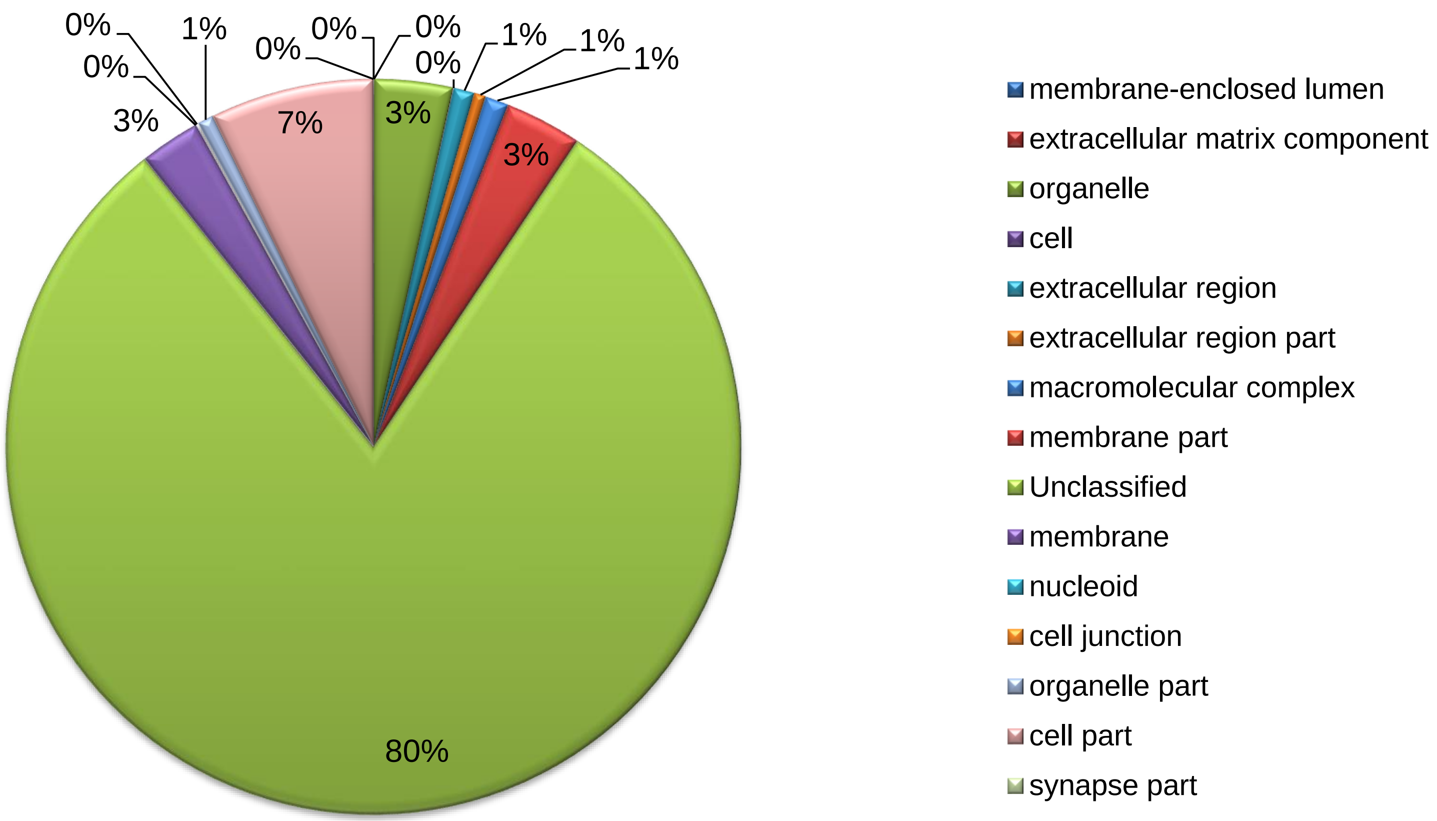

Cellular component

Supplementary figure 1a

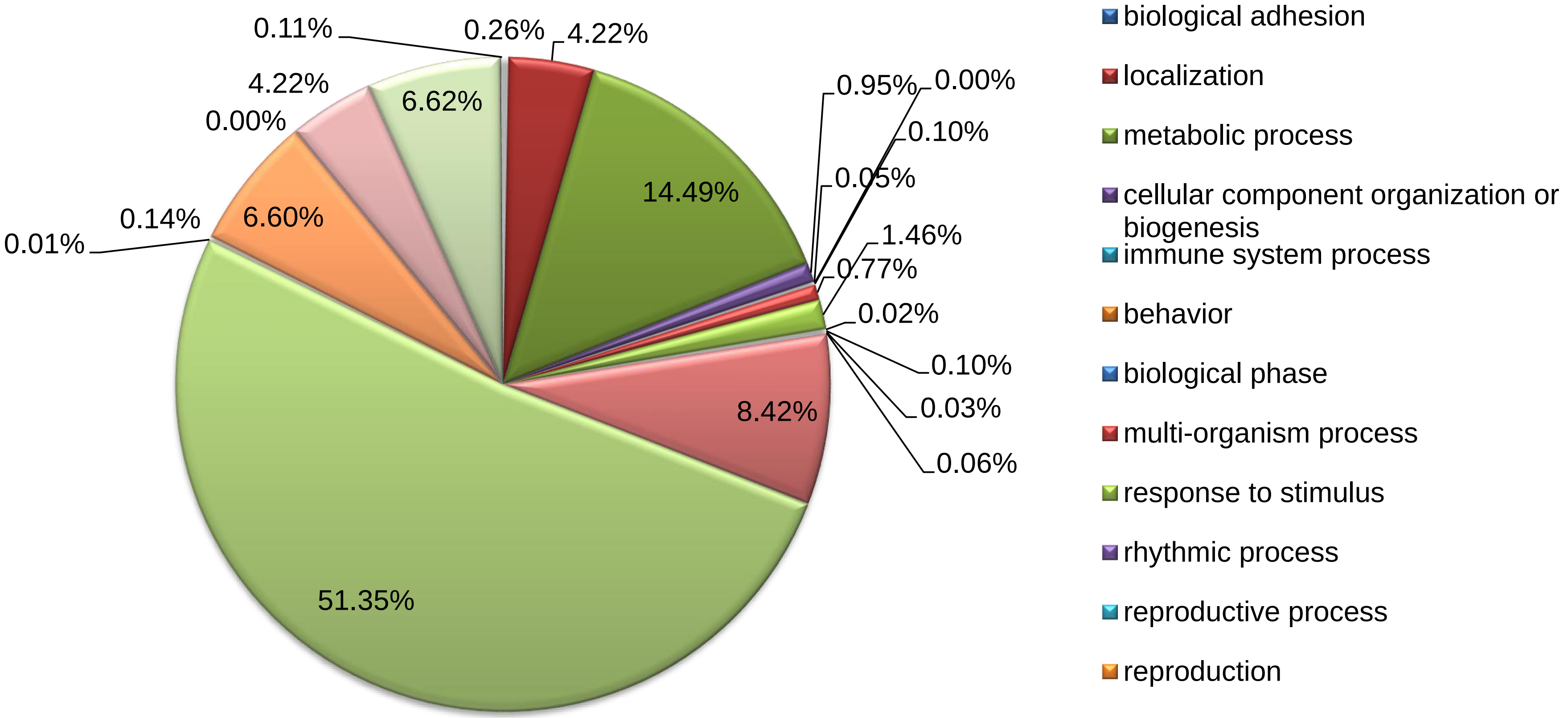

Biological process

Supplementary figure 1b

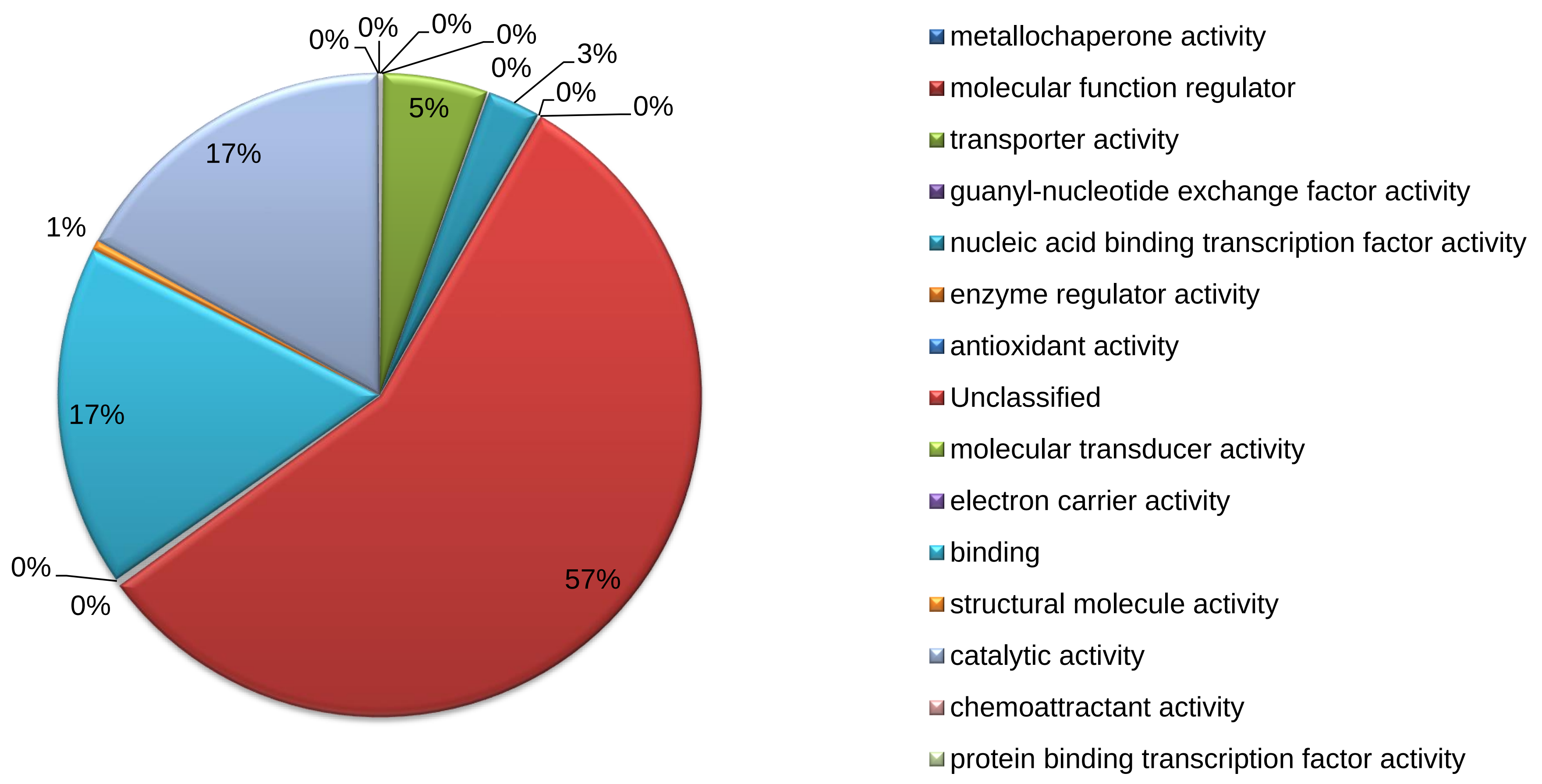

Molecular function

Supplementary figure 1c
