# Supplementary material for: De novo transcriptome assembly associated with fumonisin production by the rice pathogen Fusarium fujikuroi
Source: Data Brief. 2018 Mar 6;18:35–9. doi: 10.1016/j.dib.2018.02.072 (PMC5996136; doi:10.1016/j.dib.2018.02.072)
Supplement: Supplementary file 3 — Supplementary material [file mmc3.docx]

Data in Brief

Electronic Supplementary Material

*De novo* transcriptome assembly associated with fumonisin production by the rice pathogen *Fusarium fujikuroi*

Keerthi S. Guruge, Ryuichi Uegaki

Supplementary Table 1. List of differentially regulated genes between fumonisin-producing versus non-fumonisin-producing (control) culture conditions associated with fumonisin production (Data are given for known and putative genes)

| Upregulation (> 2-fold) | | | Downregulation (> 2-fold) | | |
| --- | --- | --- | --- | --- | --- |
| Probe name | Gene name | Fold change | Probe name | Gene name | Fold change |
| c9193_g1_i1 | putative aldehyde reductase | 97.4 | c8624_g1_i1 | PHO89-Na+/phosphate co-transporter | -360.1 |
| c6604_g1_i1 | putative sugar transport protein STL1 | 80.8 | c5431_g1_i2 | bikaverin cluster-efflux pump | -119.5 |
| c11101_g1_i1 | isoamyl alcohol oxidase | 50.0 | c8809_g1_i1 | flavoprotein | -112.1 |
| c3740_g1_i2 | L-lactate dehydrogenase (cytochrome) | 41.8 | c2068_g1_i2 | PMR1-Ca++-transporting P-type ATPase located in Golgi | -88.2 |
| c18232_g1_i1 | alpha-L-arabinofuranosidase 1 | 39.5 | c11243_g1_i1 | dopamine-responsive protein | -78.2 |
| c707_g1_i1 | putative brt1 protein | 35.2 | c15919_g1_i1 | GPM1-Phosphoglycerate mutase | -67.3 |
| c13306_g1_i1 | putative catechol O-methyltransferase | 27.5 | c5431_g1_i1 | bikaverin cluster-transcription factor | -59.8 |
| c6537_g1_i1 | glucosidase II, alpha subunit | 26.3 | c18007_g1_i1 | n-alkane-inducible cytochrome P450 | -59.1 |
| c6678_g1_i1 | amidohydrolase family protein | 24.9 | c7194_g1_i1 | fusarin C cluster-oxidoreductase | -57.9 |
| c8869_g1_i1 | 3-isopropylmalate dehydrogenase | 24.8 | c15657_g1_i1 | putative ZRT2-Zinc transporter II | -47.1 |
| c7645_g1_i1 | putative unsaturated glucuronyl hydrolase | 23.6 | c13327_g1_i1 | transcription co-repressor GAL80 | -46.8 |
| c9036_g1_i1 | alpha-glucoside transport protein | 22.1 | c6426_g1_i1 | bikaverin cluster-transcription factor enhancer | -41.2 |
| c11260_g1_i1 | related to SER33 3-phosphoglycerate dehydrogenase [ IMI 58289] | 21.1 | c7226_g1_i1 | phosphate transport protein | -37.1 |
| c6801_g1_i1 | putative L-alanine-DL-glutamate epimerase s of enolase superfamily | 20.1 | c9546_g1_i1 | N-hydroxyarylamine sulfotransferase | -35.7 |
| c4740_g1_i2 | related to integral membrane protein PTH11 [ IMI 58289] | 19.9 | c2376_g1_i1 | light induced alcohol dehydrogenase Bli-4 | -35.3 |
| c8910_g1_i1 | members of the aldo/keto reductase family | 18.6 | c12253_g1_i1 | acid phosphatase | -33.7 |
| c17777_g1_i1 | choline permease | 18.0 | c643_g1_i1 | chitin synthase/hyaluronan synthase (glycosyltransferase) | -31.9 |
| c13468_g1_i1 | related to nicotinamide mononucleotide permease [ IMI 58289] | 17.4 | c13286_g1_i1 | cytosine/adenosine deaminase | -25.3 |
| c4859_g1_i1 | putative endoglucanase IV precursor | 17.0 | c11041_g1_i1 | fusarin C cluster-hydrolase | -25.2 |
| c7544_g1_i1 | trichothecene 3-O-acetyltransferase | 16.7 | c11158_g1_i1 | fusarin C cluster-transporter | -22.5 |
| c13418_g1_i1 | 2`-O-ribosyl phosphate transferase RIT1 | 13.5 | c2766_g1_i1 | peptidylarginine deiminase s | -20.6 |
| c1837_g1_i1 | putative xylitol dehydrogenase | 13.3 | c3836_g1_i1 | URE2-nitrogen catabolite repression regulator | -20.2 |
| c11112_g1_i1 | lipase | 12.8 | c4116_g1_i3 | multidrug resistance protein | -17.9 |
| c11361_g1_i1 | endochitinase 2 precursor | 11.9 | c11630_g1_i1 | toxD protein | -17.8 |
| c13413_g1_i1 | YVC1-vacuolar cation channel | 11.8 | c3321_g1_i2 | calcium-binding protein caleosin | -17.6 |
| c13323_g1_i1 | putative CyPBP37 protein (protein binding to CyP41) | 11.4 | c16322_g1_i1 | metalloprotease MEP1 | -16.4 |
| c15647_g1_i1 | putative DIP5-glutamate and aspartate permease | 11.1 | c141_g1_i2 | TEA1-TY1 enhancer activator | -16.2 |
| c1256_g1_i2 | related to emopamil-binding protein [ IMI 58289] | 11.1 | c18572_g1_i1 | sugar transporter | -15.2 |
| c3796_g1_i1 | HXT3-Low-affinity hexose facilitator | 10.9 | c9014_g1_i1 | putative O-acetylhomoserine (thiol)-lyase | -14.8 |
| c4792_g1_i1 | NAAP-1 amino acid permease NAAP1 | 10.9 | c15732_g1_i1 | probable UGA2-succinate semialdehyde dehydrogenase [ IMI 58289] | -14.7 |
| c6555_g1_i1 | lactate 2-monooxygenase | 10.8 | c3893_g1_i1 | Protein indc11 | -14.7 |
| c15547_g1_i1 | allantoate transporter | 10.5 | c1616_g1_i2 | integral membrane protein PTH11 | -13.9 |
| c13631_g1_i1 | putative ammonium transporter MEAA | 10.3 | c15720_g1_i1 | putative galactose oxidase | -13.8 |
| c17913_g1_i1 | putative UGA2-succinate semialdehyde dehydrogenase | 10.2 | c3867_g1_i2 | fusarin C cluster-peptidase | -13.3 |
| c16358_g1_i1 | peroxisomal short-chain alcohol dehydrogenase | 10.2 | c2974_g1_i2 | monophenol monooxygenase | -13.0 |
| c587_g1_i1 | monosaccharide transporter | 10.0 | c2496_g1_i1 | aldo-keto reductase family protein | -12.6 |
| c680_g1_i1 | putative tartrate transporter | 9.7 | c18300_g1_i1 | putative beta-glucosidase | -12.1 |
| c7046_g1_i1 | putative phytoene dehydrogenase AL-1 (carotenoid biosynthesis protein al-1) | 9.7 | c9129_g1_i1 | aurofusarin/rubrofusarin efflux pump AFLT | -12.0 |
| c4792_g2_i1 | putative amino acid permease NAAP1 | 9.4 | c11132_g1_i1 | ZRT1 Zinc transporter I | -11.9 |
| c1174_g1_i1 | alpha-L-arabinofuranosidase A precursor | 8.8 | c8899_g1_i1 | aliphatic nitrilase | -11.7 |
| c15522_g1_i1 | O-methylsterigmatocystin oxidoreductase | 8.8 | c5025_g1_i2 | formate transport protein | -10.9 |
| c8775_g1_i1 | allantoate permease | 8.7 | c3609_g1_i1 | related to glycerate-and formate-dehydrogenases [ IMI 58289] | -10.7 |
| c6851_g1_i1 | putative alpha-glucoside transport protein | 8.5 | c8678_g1_i1 | endochitinase | -10.0 |
| c5034_g2_i1 | sporozoite surface protein 2 precursor | 8.3 | c16304_g1_i1 | integral membrane protein | -9.8 |
| c13691_g1_i1 | D-mandelate dehydrogenase | 7.9 | c6609_g1_i1 | low-affinity hexose transporter HXT3 | -9.5 |
| c13501_g1_i1 | putative glutamate dehydrogenase (NADP+) | 7.7 | c3757_g1_i1 | putative glutathione S-transferase | -9.5 |
| c13587_g1_i1 | proteoglycan | 7.7 | c6389_g1_i1 | putative NADPH2 dehydrogenase chain OYE2 | -9.1 |
| c11038_g1_i1 | DUF1295 domain protein | 7.6 | c6871_g1_i1 | sulfatase | -9.0 |
| c6673_g1_i1 | FRE1-ferric (and cupric) reductase | 7.3 | c3314_g1_i1 | dipeptidyl aminopeptidase B | -8.9 |
| c11552_g1_i1 | lincomycin-condensing protein lmbA | 7.2 | c1004_g1_i2 | putative reductase RED1 | -8.9 |
| c8732_g1_i1 | related to fructosyl amino acid oxidase [ IMI 58289] | 7.2 | c17821_g1_i1 | fusarin C cluster-polyketide synthase/NRPS | -8.7 |
| c8778_g1_i1 | putative sulfate permease II | 7.1 | c18107_g1_i1 | IQ calmodulin-binding motif protein | -8.5 |
| c9182_g1_i1 | probable translation machinery-associated protein 20 [ IMI 58289] | 6.9 | c5287_g1_i1 | oxidoreductase CipA-like | -8.4 |
| c15852_g1_i1 | putative D-xylose reductase | 6.8 | c2664_g1_i2 | related to extracellular cellulase CelA/allergen Asp F7-like, putative [ IMI 58289] | -8.1 |
| c855_g1_i1 | putative developmental regulator flbA | 6.7 | c17720_g1_i1 | probable type I polyketide synthase [ IMI 58289] | -8.0 |
| c16263_g1_i1 | probable endopolygalacturonase [ IMI 58289] | 6.6 | c2575_g1_i1 | related to SRP40-suppressor of mutant AC40 of RNA polymerase I and III [ IMI 58289] | -8.0 |
| c15694_g1_i1 | putative phosphogluconate dehydrogenase (decarboxylating) | 6.5 | c9031_g1_i1 | arylamine N-acetyltransferase | -7.9 |
| c8647_g1_i1 | 2-deoxy-D-gluconate 3-dehydrogenase | 6.3 | c5117_g1_i1 | short-chain alcohol dehydrogenase | -7.9 |
| c13275_g1_i1 | L-lactate dehydrogenase | 6.2 | c17769_g1_i1 | cyanovirin-N family protein | -7.9 |
| c13343_g1_i1 | putative PYC2 Pyruvate carboxylase 2 | 6.2 | c2003_g1_i2 | ankyrin repeat protein | -7.6 |
| c10928_g1_i1 | related to P. aeruginosa hyuA and hyuB [ IMI 58289] | 6.2 | c2636_g2_i1 | myo-inositol transport protein ITR1 | -7.4 |
| c13870_g1_i1 | putative GAP1-General amino acid permease | 6.1 | c17996_g1_i1 | carboxylesterase | -7.4 |
| c18342_g1_i1 | putative fumarylacetoacetate hydrolase | 6.0 | c3210_g1_i2 | acid phosphatase precursor (pH 6-optimum acid phosphatase) | -7.4 |
| c97_g1_i1 | regulatory protein amdA | 5.8 | c960_g1_i1 | isotrichodermin C-15 hydroxylase (cytochrome P-450 monooxygenase CYP65A1) | -7.2 |
| c4577_g1_i1 | putative TIF6-translation initiation factor 6 (eIF6) | 5.8 | c3406_g1_i1 | DUF1295 domain protein | -7.0 |
| c3213_g1_i1 | several transaminase | 5.7 | c18371_g1_i1 | putative endothiapepsin precursor | -7.0 |
| c15722_g1_i1 | putative UGA1-4-aminobutyrate aminotransferase (GABA transaminase) | 5.6 | c17801_g1_i1 | opsin-like protein | -6.9 |
| c13626_g1_i1 | quinate utilization oxidoreductase QutH | 5.6 | c13473_g1_i1 | peptidase yuxL | -6.9 |
| c4067_g1_i2 | putative multidrug transporter | 5.5 | c15636_g1_i1 | glucan 1,3-beta-glucosidase | -6.9 |
| c10108_g1_i1 | ARO1-arom pentafunctional enzyme | 5.3 | c8702_g1_i1 | epoxide hydrolase | -6.9 |
| c4024_g1_i2 | Metal tolerance protein 7 | 5.1 | c10060_g1_i1 | 2,4-dienoyl-CoA reductase precursor | -6.9 |
| c11063_g1_i1 | related to HRT2-high level expression reduced Ty3 transposition [ IMI 58289] | 5.1 | c18195_g1_i1 | trihydrophobin precursor | -6.8 |
| c15886_g1_i1 | transaldolase | 5.1 | c4336_g1_i1 | small s protein | -6.8 |
| c2644_g1_i2 | integral membrane protein pth11 | 5.0 | c3998_g1_i2 | histone acetyltransferase subunit HAT1 | -6.7 |
| c11428_g1_i1 | exoenzymes regulatory protein aepA precursor | 5.0 | c11481_g1_i1 | related to tol protein [ IMI 58289] | -6.7 |
| c899_g1_i1 | fumarate reductase | 5.0 | c4084_g2_i1 | putative DFG5 protein | -6.7 |
| c994_g1_i1 | putative neutral amino acid permease | 4.9 | c3731_g1_i1 | coenzyme a synthetase | -6.6 |
| c17756_g1_i1 | ABC transporter protein (ATP-binding-cassette protein) | 4.9 | c16045_g1_i1 | oxidoreductase, FAD-binding | -6.4 |
| c2356_g1_i1 | G protein coupled receptor like protein | 4.8 | c11886_g1_i1 | putative alpha/beta fold family hydrolase | -6.3 |
| c18438_g1_i1 | HOL1-putative substrate-H+ antiporter-unknown biological function | 4.7 | c11306_g1_i1 | putative nitrite reductase | -6.3 |
| c2046_g1_i1 | Dal5p | 4.6 | c6944_g1_i1 | putative glucan 1,4-alpha-glucosidase | -6.3 |
| c12009_g1_i1 | putative starvation sensing protein rspA | 4.6 | c3202_g1_i1 | 2`-hydroxyisoflavone reductase | -6.3 |
| c8676_g1_i1 | putative 2-methylcitrate dehydratase | 4.6 | c8908_g1_i1 | thioredoxin | -6.3 |
| c11056_g1_i1 | putative peroxisomal amine oxidase (copper-containing) | 4.6 | c17677_g1_i1 | ATP-binding multidrug cassette transport protein | -6.2 |
| c17904_g1_i1 | putative acetoacetyl-CoA thiolase | 4.6 | c6060_g1_i2 | general RNA polymerase II transcription factor TAF12 | -6.2 |
| c10959_g1_i1 | putative PRX1-mitochondrial isoform of thioredoxin peroxidase | 4.5 | c3666_g1_i2 | Isoflavone reductase P3 | -6.1 |
| c16147_g1_i1 | SUC2-invertase (sucrose hydrolyzing enzyme) | 4.5 | c6418_g1_i1 | related to dihydrodipicolinate synthetase [ IMI 58289] | -6.0 |
| c4452_g1_i2 | NIT3 Nitrilase | 4.4 | c13254_g1_i1 | nonribosomal peptide synthetase MxcG (component of the myxochelin iron transport regulon) | -6.0 |
| c13671_g1_i1 | cytochrome P450 monooxygenase | 4.4 | c1843_g1_i1 | ACB 4-hydroxyacetophenone monooxygenase | -6.0 |
| c2123_g1_i2 | monocarboxylate transporter 2 | 4.3 | c15700_g1_i1 | neutral amino acid permease | -5.9 |
| c3474_g1_i1 | putative glutamine synthetase | 4.3 | c5131_g1_i3 | sentrin-specific protease SENP8 (SUMO-specific protease) | -5.9 |
| c9433_g1_i1 | negative-acting regulatory protein | 4.3 | c13303_g1_i1 | putative Diphthamide biosynthesis protein 3 | -5.9 |
| c9324_g1_i1 | AMP-binding protein | 4.2 | c15573_g1_i1 | cell wall glycoprotein | -5.8 |
| c13738_g1_i1 | related to beta-galactosidase [ IMI 58289] | 4.2 | c7561_g1_i1 | N-acetylglucosamine-6-phosphate deacetylase | -5.8 |
| c9646_g1_i1 | UNR-interacting protein STRAP (serine-threonine kinase receptor-associated protein) | 4.2 | c11248_g1_i1 | oxidoreductase | -5.8 |
| c15660_g1_i1 | KES1-involved in ergosterol biosynthesis | 4.2 | c7132_g1_i1 | related to novobiocin biosynthesis protein novR [ IMI 58289] | -5.7 |
| c13389_g1_i1 | putative DAL7-malate synthase 2 | 4.2 | c4228_g1_i1 | related to GTPase Rho [ IMI 58289] | -5.7 |
| c11376_g1_i1 | putative D-lactate dehydrogenase (cytochrome) | 4.2 | c16475_g1_i1 | putative trichothecene biosynthesis protein | -5.7 |
| c4260_g1_i1 | permease-unknown function | 4.1 | c2380_g1_i1 | BCS1 protein precursor | -5.7 |
| c13767_g1_i1 | IST2 protein | 4.1 | c9364_g1_i1 | phenol 2-monooxygenase | -5.6 |
| c11685_g1_i1 | putative sulfate adenylyltransferase | 4.1 | c16118_g1_i1 | endo-1,3-beta-glucanase | -5.6 |
| c17738_g1_i1 | endoglucanase B | 4.1 | c11217_g1_i1 | Tripeptidyl-peptidase I precursor | -5.5 |
| c6972_g1_i1 | putative prolidase (Xaa-Pro dipeptidase) | 4.0 | c667_g1_i2 | choline kinase | -5.5 |
| c709_g2_i1 | putative transcription activator protein acu-15 | 4.0 | c4114_g1_i1 | class V chitinase | -5.4 |
| c6210_g1_i1 | atrophin-1 | 4.0 | c9380_g1_i1 | arabinan endo-1,5-alpha-L-arabinosidase A precursor | -5.4 |
| c5549_g1_i2 | transcription factor TamA | 4.0 | c5891_g1_i2 | ankyrin | -5.4 |
| c1840_g1_i2 | carotene cyclase | 4.0 | c4848_g2_i1 | putative pectate lyase | -5.2 |
| c3309_g1_i1 | DUF1237 domain protein | 4.0 | c10983_g1_i1 | putative glutamate decarboxylase | -5.2 |
| c15619_g1_i1 | pyruvate dehydrogenase (lipoamide) alpha chain precursor | 4.0 | c16466_g1_i1 | haloalkanoic acid dehalogenase | -5.2 |
| c1901_g1_i1 | putative beta karyopherin | 4.0 | c14427_g1_i1 | salicylate hydroxylase | -5.1 |
| c11522_g1_i1 | dehydroshikimate dehydratase | 3.9 | c12170_g1_i1 | ABC transporter | -5.1 |
| c4063_g1_i2 | phosphatase 2a inhibitor | 3.9 | c15994_g1_i1 | LSB3-possible role in the regulation of actin cytoskeletal organization | -5.1 |
| c9078_g1_i1 | C2H2 finger domain protein | 3.9 | c11741_g1_i1 | putative maltase | -5.0 |
| c6582_g1_i1 | putative transcriptional regulator | 3.9 | c3928_g2_i2 | putative flavohemoglobin | -5.0 |
| c1840_g1_i1 | putative geranylgeranyl-diphosphate geranylgeranyltransferase (AL-2) | 3.9 | c5931_g1_i3 | AP-1-like transcription factor | -4.9 |
| c16320_g1_i1 | sulfate transporter protein | 3.9 | c5079_g1_i2 | triacylglycerol lipase II precursor | -4.9 |
| c13326_g1_i1 | probable glycine hydroxymethyltransferase [ IMI 58289] | 3.9 | c16071_g1_i1 | beta-1,4-mannosyl-glycoprotein 4-beta-N-acetylglucosaminyltransferase | -4.8 |
| c17852_g1_i1 | putative PHO8-repressible alkaline phosphatase vacuolar | 3.9 | c3017_g1_i1 | putative NADH-ubiquinone oxidoreductase subunit, mitochondrial precursor | -4.8 |
| c5443_g1_i1 | tetracycline resistance protein TCR1 | 3.9 | c18358_g1_i1 | putative carnitine transporter | -4.8 |
| c4452_g1_i1 | Zn(II)2Cys6 transcriptional activator | 3.9 | c17729_g1_i1 | low affininty zinc transporter | -4.7 |
| c12148_g1_i1 | putative fusarubin cluster-esterase | 3.8 | c2233_g1_i1 | putative thioredoxin | -4.7 |
| c5782_g1_i1 | hydroxyproline-rich glycoprotein precursor | 3.8 | c17674_g1_i1 | putative rAsp f 9 allergen | -4.7 |
| c13725_g1_i1 | protein CTF18 | 3.8 | c4007_g1_i2 | sulfur controller-2 protein | -4.6 |
| c11069_g1_i1 | putative mitochondrial substrate carrier | 3.8 | c18651_g1_i1 | putative Cyanamide hydratase | -4.6 |
| c9101_g1_i1 | putative glutamine-tRNA ligase | 3.7 | c2639_g1_i1 | zinc transporter | -4.6 |
| c8811_g1_i1 | putative delta(24)-sterol c-methyltransferase (ERG6) | 3.7 | c16513_g1_i1 | putative iron-dependent peroxidase | -4.6 |
| c349_g1_i1 | related to cell cycle progression protein [ IMI 58289] | 3.7 | c11722_g1_i1 | aldehyde reductase II | -4.5 |
| c4528_g2_i1 | alcohol dehydrogenase | 3.7 | c4743_g1_i1 | excitatory amino acid transporter | -4.5 |
| c4086_g1_i1 | QA-1F quinic acid utilization activator QA-1F | 3.7 | c3049_g1_i1 | putative benzoate 4-monooxygenase cytochrome P450 | -4.5 |
| c13659_g1_i1 | aminomethyltransferase precursor (glycine cleavage system protein T) | 3.7 | c5956_g2_i1 | aspartic proteinase OPSB | -4.4 |
| c17839_g1_i1 | putative zinc finger protein ZPR1 | 3.7 | c14189_g1_i1 | pirin | -4.3 |
| c2364_g1_i2 | FCP1-TFIIF interacting component of CTD phosphatase | 3.7 | c18609_g1_i1 | related to monooxigenase [ IMI 58289] | -4.3 |
| c6775_g1_i1 | GNAT family acetyltransferase | 3.7 | c2205_g1_i2 | YTP1 protein | -4.3 |
| c4542_g1_i1 | TPR domain protein | 3.7 | c9397_g1_i1 | putative 3-ketosteroid-delta-1-dehydrogenase | -4.3 |
| c2079_g1_i1 | WD repeat protein | 3.7 | c2332_g1_i1 | putative MUS-38 protein, involved in nucleotide excision repair | -4.3 |
| c17687_g1_i1 | putative alpha-glucosidase (maltase) | 3.7 | c6115_g1_i2 | hydrolase or acyltransferase (alpha/beta hydrolase superfamily) | -4.2 |
| c3999_g2_i1 | putative isocitrate lyase (acu-3) | 3.7 | c13666_g1_i1 | ADH3-alcohol dehydrogenase III | -4.2 |
| c8627_g1_i1 | glycerol-3-phosphate dehydrogenase precursor | 3.6 | c921_g1_i1 | heterokaryon incompatibility protein het-6 | -4.1 |
| c11148_g1_i1 | putative methylcrotonyl-CoA carboxylase beta chain, mitochondrial precursor | 3.6 | c8769_g1_i1 | trans-aconitate 3-methyltransferase | -4.1 |
| c17573_g1_i1 | aryl-alcohol dehydrogenase | 3.6 | c6840_g1_i1 | SPR1-exo-1,3-beta-glucanase precursor | -4.1 |
| c2116_g1_i1 | related to DHA14-like major facilitator efflux transporter (MFS transporter) [ IMI 58289] | 3.6 | c5491_g1_i2 | nitrate assimilation regulatory protein nirA | -4.1 |
| c6269_g2_i1 | chitinase | 3.6 | c5662_g1_i1 | related to fluconazole resistance protein [ IMI 58289] | -4.0 |
| c1850_g1_i1 | related to GS1 protein [ IMI 58289] | 3.6 | c6541_g1_i1 | cutinase transcription factor 1 beta | -4.0 |
| c10380_g1_i1 | acetyltransferase | 3.6 | c6422_g1_i1 | nif-specific regulatory protein | -4.0 |
| c8688_g1_i1 | putative drug facilitator PEP5 | 3.6 | c11671_g1_i1 | DNA repair protein NTG1 | -3.9 |
| c11180_g1_i1 | CLC chloride channel protein | 3.6 | c11350_g1_i1 | related to beta transducin-like protein [ IMI 58289] | -3.9 |
| c15714_g1_i1 | putative glutamate dehydrogenase, NAD(+)-specific | 3.5 | c5005_g1_i4 | related to methyltransferase [ IMI 58289] | -3.9 |
| c8726_g1_i1 | putative chorismate synthase/flavin reductase, NADPH-dependent | 3.5 | c8625_g1_i1 | fatty acid hydroxylase | -3.8 |
| c8651_g1_i1 | putative maleylacetoacetate isomerase | 3.5 | c2075_g1_i1 | putative aflatoxin efflux pump AFLT | -3.8 |
| c10968_g1_i1 | HNM1-Choline permease | 3.5 | c15836_g1_i1 | putative vacuolar iron transport protein | -3.8 |
| c278_g1_i1 | putative kinesin-related protein bimC | 3.5 | c7355_g1_i1 | putative metallo-beta-lactamase domain protein | -3.8 |
| c13673_g1_i1 | alanine racemase | 3.5 | c5771_g1_i2 | putative sterigmatocystin biosynthesis lipase/esterase STCI | -3.8 |
| c858_g1_i1 | putative nuclear export sequence-containing nonribosomal protein | 3.5 | c15525_g1_i1 | endo-beta-1,4-glucanase | -3.7 |
| c5933_g2_i3 | Mg2 transporter protein, CorA-like | 3.5 | c11289_g1_i1 | T10 protein | -3.7 |
| c13866_g1_i1 | putative aspartate aminotransferase, cytoplasmic | 3.5 | c3809_g1_i1 | putative CYB2-lactate dehydrogenase cytochrome b2 | -3.7 |
| c13838_g1_i1 | polysaccharide synthase Cps1 | 3.5 | c4674_g1_i1 | putative potassium transporter hak-1 | -3.7 |
| c16145_g1_i1 | ubiquitin thiolesterase | 3.4 | c4476_g1_i4 | YPC1-Alkaline Ceramidase | -3.7 |
| c10953_g1_i1 | cysteinyl-tRNA synthetase | 3.4 | c3688_g1_i1 | haloacetate dehalogenase H-1 | -3.7 |
| c12177_g1_i1 | probable pectin lyase precursor [ IMI 58289] | 3.4 | c15733_g1_i1 | polypeptide chain release factor | -3.7 |
| c18155_g1_i1 | stress protein | 3.4 | c4804_g1_i1 | 6-hydroxy-d-nicotine oxidase | -3.7 |
| c2248_g1_i1 | alpha-galactosidase precursor | 3.4 | c4540_g1_i2 | putative NADPH reductase | -3.6 |
| c16181_g1_i1 | aldehyde dehydrogenase | 3.4 | c4970_g1_i1 | protein-arginine deiminase type II | -3.6 |
| c9189_g1_i1 | putative translation elongation factor eEF4 | 3.4 | c18132_g1_i1 | putative Maltose permease | -3.6 |
| c197_g1_i1 | ubiquitin-conjugating protein | 3.4 | c11456_g1_i1 | acid proteinase PEPI precursor | -3.6 |
| c102_g1_i1 | Het-c protein | 3.4 | c4553_g1_i3 | arginine-tRNA-protein transferase | -3.5 |
| c5395_g2_i3 | MVP1 protein | 3.4 | c1527_g1_i1 | nucleoside-diphosphate-sugar epimerase | -3.5 |
| c1788_g1_i1 | cysteine synthase B | 3.4 | c2787_g1_i1 | phospholipase C | -3.5 |
| c3901_g2_i1 | helicase of the Snf2/Rad54 family | 3.4 | c14783_g1_i1 | SER3-3-phosphoglycerate dehydrogenase | -3.5 |
| c6796_g1_i2 | choline dehydrogenase | 3.4 | c13317_g1_i1 | 3-oxoadipate enol-lactonase I | -3.5 |
| c5859_g1_i1 | putative isocitrate dehydrogenase | 3.3 | c16494_g1_i1 | HPP family protein | -3.5 |
| c4884_g1_i1 | RNA-binding protein ous to eukaryotic snRNP | 3.3 | c5560_g1_i2 | WSC4-Cell wall integrity and stress response component 4 | -3.5 |
| c9350_g1_i1 | putative ribonuclease H | 3.3 | c5201_g1_i1 | transcriptional regulator atrx | -3.5 |
| c13433_g1_i1 | hypothetical protein LW94_6398 | 3.3 | c1501_g1_i1 | cystathionine gamma-synthase | -3.4 |
| c1161_g2_i1 | lipid binding protein Tfs1p | 3.3 | c6665_g1_i1 | putative alternative oxidase precursor, mitochondrial | -3.4 |
| c18500_g1_i1 | peroxisomal amine oxidase (copper-containing) | 3.3 | c2294_g1_i2 | aromatic-L-amino-acid decarboxylase | -3.4 |
| c2771_g1_i1 | putative parvulin (peptidylprolyl isomerase) | 3.3 | c2737_g2_i1 | CPS1-Gly-X carboxypeptidase YSCS precursor | -3.4 |
| c2949_g1_i2 | putative purine nucleoside permease | 3.3 | c13739_g1_i1 | acyl-CoA thiolesterase | -3.4 |
| c11064_g1_i1 | ferric reductase Fre2p | 3.3 | c6350_g2_i3 | zinc transporter | -3.4 |
| c9722_g1_i1 | trans-aconitate 2-methyltransferase | 3.3 | c14069_g1_i1 | D-lactate dehydrogenase | -3.4 |
| c5446_g1_i2 | putative YCS4-subunit of condensin protein complex | 3.3 | c6394_g1_i1 | triose phosphate/3-phosphoglycerate/phosphate translocator | -3.3 |
| c255_g1_i1 | triacylglycerol lipase | 3.3 | c17751_g1_i1 | PPN1-vacuolar endopolyphosphatase | -3.3 |
| c11305_g1_i1 | regulatory protein alcR | 3.3 | c3444_g1_i1 | DAL1-Allantoinase | -3.3 |
| c14633_g1_i1 | BNR/Asp-box repeat domain protein | 3.3 | c15597_g1_i1 | probable ABC1 transport protein [ IMI 58289] | -3.3 |
| c18658_g1_i1 | bystin | 3.3 | c6580_g1_i1 | TRI15-putative transcription factor | -3.2 |
| c10318_g1_i1 | related to ARCA protein [ IMI 58289] | 3.3 | c2482_g1_i2 | related to NUDIX domain [ IMI 58289] | -3.2 |
| c5951_g1_i1 | protein LAC1 | 3.3 | c13836_g1_i1 | alpha-amylase A precursor | -3.2 |
| c12714_g1_i1 | GNAT family acetyltransferase | 3.3 | c14138_g1_i1 | cation diffusion facilitator 10 | -3.2 |
| c6760_g1_i1 | vacuolar Ca2+/H+ antiporter | 3.3 | c13448_g1_i1 | nik-1 protein (Os-1p protein) | -3.2 |
| c15635_g1_i1 | putative Modin | 3.2 | c6178_g1_i1 | WSS1 Protein involved in sister chromatid separation and segregation | -3.2 |
| c5408_g1_i1 | putative Na+-transporting ATPase ENA-1 (sodium P-type ATPase ENA-1) | 3.2 | c14970_g1_i1 | related to prenyl cysteine carboxyl methyltransferase [ IMI 58289] | -3.2 |
| c13685_g1_i1 | Amid-like NADH oxidoreductase | 3.2 | c3389_g1_i1 | putative mfs-multidrug-resistance transporter | -3.2 |
| c4290_g1_i2 | COQ2-para-hydroxybenzoate--polyprenyltransferase | 3.2 | c4753_g1_i2 | Zn2Cys6 binuclear cluster DNA-binding-like protein | -3.2 |
| c11266_g1_i1 | putative U1 snRNP protein | 3.2 | c3663_g1_i1 | glutathione S-transferase GST-6.0 | -3.2 |
| c3774_g1_i2 | putative amino acid transporter | 3.2 | c9272_g1_i1 | trichothecene biosynthesis protein | -3.2 |
| c10944_g1_i1 | gamma-tubulin complex component | 3.2 | c4610_g1_i2 | putative alpha-1,2-mannosyltransferase | -3.1 |
| c16474_g1_i1 | thiosulfate sulfurtransferase | 3.2 | c6195_g1_i2 | PTR2-Di-and tripeptide permease | -3.1 |
| c3766_g1_i1 | tricarboxylate carrier | 3.2 | c13730_g1_i1 | putative glycerol-3-phosphate dehydrogenase (NAD) | -3.1 |
| c18171_g1_i1 | Rtm1p | 3.2 | c6845_g1_i1 | protein BTN1 | -3.1 |
| c798_g1_i1 | putative hnRNP arginine N-methyltransferase | 3.2 | c15971_g1_i1 | putative phosphate transport protein MIR1 | -3.1 |
| c1478_g1_i1 | krueppel protein | 3.2 | c13528_g1_i1 | zinc alcohol dehydrogenase | -3.1 |
| c2924_g1_i2 | putative RIO2 protein | 3.2 | c6185_g1_i3 | putative mago nashi protein | -3.1 |
| c11173_g1_i1 | putative DNA replication licensing factor (nimQ) | 3.1 | c4966_g1_i2 | putative homoserine O-acetyltransferase | -3.1 |
| c18764_g1_i1 | agmatinase | 3.1 | c5573_g1_i3 | dis1-suppressing protein kinase dsk1 | -3.1 |
| c10949_g1_i1 | putative heat shock protein | 3.1 | c1437_g1_i2 | branched-chain amino acid aminotransferase | -3.1 |
| c11895_g1_i1 | putative HTS1-histidine--tRNA ligase, mitochondrial | 3.1 | c6378_g1_i15 | related to bifunctional GTP cyclohydrolase II/3, 4-dihydroxy-2butanone-4-phosphate synthase [ IMI 58289] | -3.1 |
| c6621_g1_i1 | putative methionyl aminopeptidase | 3.1 | c9131_g1_i1 | adenosine triphosphate (ATP) synthase subunit 6, partial (mitochondrion) | -3.0 |
| c6854_g1_i1 | putative general amino acid permease | 3.1 | c17867_g1_i1 | C4-dicarboxylate transport protein mae1 | -3.0 |
| c5462_g2_i2 | origin recognition complex subunit 4 | 3.1 | c2671_g1_i2 | putative uracil permease | -3.0 |
| c9214_g1_i1 | putative regulatory subunit for Glc7p | 3.1 | c5163_g1_i1 | KEL2-involved in cell fusion and morphogenesis | -3.0 |
| c17970_g1_i1 | GPR1 protein | 3.1 | c6457_g1_i1 | putative beta(1-3)glucanosyltransferase | -3.0 |
| c881_g1_i1 | putative protein CGRA (conserved fungal nucleolar protein) | 3.1 | c3217_g1_i1 | probable argonaute like post-transcriptional gene silencing protein QDE-2 [ IMI 58289] | -3.0 |
| c14121_g1_i1 | putative CDC28-cyclin-dependent protein kinase | 3.1 | c17911_g1_i1 | cercosporin resistance protein | -3.0 |
| c16420_g1_i1 | Chromosome segregation in meiosis protein 3 | 3.1 | c1600_g1_i1 | nitrogen metabolic regulation protein nmr | -3.0 |
| c3660_g1_i1 | pescadillo development protein | 3.1 | c6338_g1_i6 | related to tripeptidyl-peptidase I [ IMI 58289] | -3.0 |
| c8759_g1_i1 | N-acetylgalactosamine-6-sulfatase precursor | 3.1 | c3623_g1_i1 | polysaccharide deacetylase | -3.0 |
| c15779_g1_i1 | exportin T (tRNA exportin) | 3.1 | c590_g1_i1 | putative endo-1,4-beta-xylanase B precursor | -3.0 |
| c3766_g1_i2 | tricarboxylate carrier | 3.0 | c11211_g1_i1 | protein kinase | -3.0 |
| c464_g1_i1 | seed maturation protein pm25 | 3.0 | c3580_g1_i1 | serine protease | -3.0 |
| c2397_g1_i1 | putative subunit of pre-replication complex | 3.0 | c8861_g1_i1 | ERO1 protein, required for protein disulfide bond formation in the ER | -2.9 |
| c15830_g1_i1 | putative histone acetyltransferase | 3.0 | c2157_g1_i2 | vacuolar zinc efflux protein | -2.9 |
| c11604_g1_i1 | putative glycosidase | 3.0 | c15872_g1_i1 | putative amino acid transport protein GAP1 | -2.9 |
| c3353_g1_i1 | mitotic spindle biogenesis protein Spc19 | 3.0 | c4943_g1_i2 | putative carnitine acetyl transferase FacC | -2.9 |
| c17711_g1_i1 | related to cell cycle checkpoint protein RAD17 [ IMI 58289] | 3.0 | c7063_g1_i1 | 1-acyldihydroxyacetone-phosphate reductase | -2.9 |
| c5775_g1_i3 | putative thiamine repressed nmt1 protein | 3.0 | c5839_g1_i1 | secretory lipase | -2.9 |
| c4101_g1_i1 | QDE3 protein | 3.0 | c15749_g1_i1 | putative lysophospholipase (lpl) | -2.9 |
| c8935_g1_i1 | Lysophospholipase | 3.0 | c11514_g1_i1 | DUF1338 domain protein | -2.9 |
| c15902_g1_i1 | putative asparaginyl-tRNA-synthetase | 3.0 | c6293_g1_i1 | related to multidrug resistance protein [ IMI 58289] | -2.9 |
| c11735_g1_i1 | allantoate transport protein | 3.0 | c8681_g1_i1 | mitochondrial integral membrane protein | -2.9 |
| c16589_g1_i1 | xylosidase/arabinosidase | 3.0 | c18242_g1_i1 | phospholipid-translocating ATPase | -2.9 |
| c6610_g1_i1 | putative 2-isopropylmalalate synthase | 3.0 | c5155_g1_i3 | putative trehalose synthase | -2.9 |
| c2291_g1_i1 | putative ECM16-putative DEAH-box RNA helicase | 3.0 | c13557_g1_i1 | CIT2-citrate (si)-synthase, peroxisomal | -2.9 |
| c1653_g1_i1 | DUF453 domain protein | 3.0 | c11001_g1_i1 | 15-hydroxyprostaglandin dehydrogenase | -2.9 |
| c7218_g1_i1 | hydroxyquinol-1,2-dioxygenase | 3.0 | c5185_g1_i1 | purine-cytosine permease | -2.8 |
| c2433_g1_i2 | putative rhamnogalacturonase A precursor | 3.0 | c17673_g1_i1 | hypothetical protein | -2.8 |
| c6571_g1_i1 | putative arginase | 3.0 | c18216_g1_i1 | POX1-acyl-CoA oxidase | -2.8 |
| c4237_g2_i1 | transcription factor | 3.0 | c11491_g1_i1 | putative LAP3-member of the GAL regulon | -2.8 |
| c6847_g1_i1 | related to dehydrogenases with different specificities (related to short-chain alcohol dehydrogenases) [ IMI 58289] | 3.0 | c6232_g1_i1 | hexamer-binding protein HEXBP | -2.8 |
| c11449_g1_i1 | neuraminidase (sialidase) | 3.0 | c11084_g1_i1 | catechol O-methyltransferase | -2.8 |
| c4179_g1_i1 | putative MBP1-transcription factor, subunit of the MBF factor | 3.0 | c1945_g1_i1 | gamma-tubulin complex component GCP4 | -2.8 |
| c4739_g1_i1 | CDC14-dual specificity phosphatase | 3.0 | c1144_g1_i2 | ADP-ribosylation factor | -2.8 |
| c13957_g1_i1 | dipeptidyl aminopeptidase/acylaminoacyl-peptidase | 3.0 | c16432_g1_i1 | related to isoamyl alcohol oxidase [ IMI 58289] | -2.8 |
| c2927_g1_i2 | putative MUP1-High affinity methionine permease | 3.0 | c17799_g1_i1 | quinone reductase | -2.8 |
| c16205_g1_i1 | GrpB domain protein | 3.0 | c8874_g1_i1 | BSC1 Transcript encoded by this ORF shows a high level of stop codon bypass | -2.8 |
| c17666_g1_i1 | putative ADP, ATP carrier protein (ADP/ATP translocase) | 3.0 | c6852_g1_i1 | UDP-galactopyranose mutase | -2.8 |
| c6365_g2_i1 | hsp70 protein | 3.0 | c6738_g1_i1 | transcriptional activator Mut3p | -2.8 |
| c1008_g1_i2 | RRP46 protein, involved in rRNA processing | 3.0 | c18532_g1_i1 | YSC84-protein involved in the organization of the actin cytoskeleton | -2.8 |
| c11705_g1_i1 | RNA binding protein Pym | 3.0 | c6903_g1_i1 | glucose/galactose transporter | -2.7 |
| c11042_g1_i1 | putative eukaryotic translation initiation factor 3 subunit 7 | 3.0 | c16132_g1_i1 | putative MNN2-type II membrane protein | -2.7 |
| c9896_g1_i1 | monoamine oxidase N | 3.0 | c5679_g1_i1 | putative DUF636 domain protein | -2.7 |
| c1931_g1_i2 | putative RHO1 protein | 2.9 | c3723_g1_i1 | KEX1 protein precursor | -2.7 |
| c14033_g1_i1 | methionine synthase II (cobalamin-independent) | 2.9 | c6122_g1_i2 | FAD dependent oxidoreductase superfamily | -2.7 |
| c6890_g1_i1 | putative cytochrome P450 51 (eburicol 14 alpha-demethylase) | 2.9 | c985_g2_i1 | DEF1-coordinates repair and RNA pol II proteolysis in response to DNA damage | -2.7 |
| c6622_g1_i1 | putative RRB1-involved in the regulation of ribosome biosynthesis | 2.9 | c9208_g1_i1 | 2-polyprenyl-6-methoxyphenol hydroxylase and related FAD-dependent oxidoreductase | -2.7 |
| c4874_g1_i1 | putative extragenic suppressor of the bimD6 mutation | 2.9 | c17221_g1_i1 | putative ribosomal protein L31.e.A, cytosolic | -2.7 |
| c6715_g1_i1 | sugar transport protein STL1 | 2.9 | c14263_g1_i1 | Protein urg3 | -2.7 |
| c17668_g1_i1 | putative WD40-repeat protein (notchless protein) | 2.9 | c4871_g1_i1 | GIP2 Glc7p-interacting protein | -2.7 |
| c8620_g1_i1 | putative heat shock protein HSP70 (regulator protein involved in pleiotropic drug resistance) | 2.9 | c4689_g2_i2 | cytosine deaminase and related metal-dependent hydrolase | -2.6 |
| c7266_g1_i1 | putative ribosomal RNA-processing protein 12 | 2.9 | c15656_g1_i1 | related to 3-phytase precursor [ IMI 58289] | -2.6 |
| c9896_g1_i1 | monoamine oxidase N | 3.0 | c7043_g1_i1 | hexokinase | -2.6 |
| c6892_g1_i1 | putative xanthine phosphoribosyl transferase | 2.9 | c3617_g1_i1 | multidrug resistance protein fnx1 | -2.6 |
| c9151_g1_i1 | lactose permease | 2.9 | c5426_g1_i1 | suppressor protein PSP1 | -2.6 |
| c13993_g1_i1 | putative calmodulin-dependent protein kinase | 2.9 | c4961_g1_i2 | putative RIC1 protein | -2.6 |
| c2159_g1_i1 | hypothetical protein LW94_14250 | 2.9 | c14243_g1_i1 | monooxygenase | -2.6 |
| c14462_g1_i1 | putative exopolygalacturonase | 2.9 | c6198_g1_i2 | related to CCC2-P-type ATPase involved in export of Cu++ from the cytosol into intracellular, secret [ IMI 58289] | -2.6 |
| c6644_g1_i1 | putative iron inhibited ABC transporter 2 | 2.9 | c1728_g1_i1 | related to zinc cluster transcription factor [ IMI 58289] | -2.6 |
| c4517_g1_i2 | putative SCRO protein | 2.9 | c11641_g1_i1 | diaminopropionate ammonia-lyase | -2.6 |
| c1740_g1_i1 | putative serine/threonine protein phosphatase-z-like | 2.9 | c485_g1_i2 | 24-dehydrocholesterol reductase precursor | -2.6 |
| c9620_g1_i1 | related to putative tartrate transporter [ IMI 58289] | 2.9 | c2343_g1_i1 | protoporphyrinogen oxidase | -2.6 |
| c6924_g1_i1 | transcription factor Ask10p | 2.9 | c15781_g1_i1 | vacuolar membrane protein HMT1 (heavy metal tolerance protein) | -2.6 |
| c16174_g1_i1 | DNA-directed RNA polymerase III | 2.9 | c5230_g1_i3 | isoleucine tRNA ligase | -2.6 |
| c93_g1_i1 | heat-and acid-stable phosphoprotein | 2.9 | c13383_g1_i1 | putative stearoyl-CoA desaturase | -2.6 |
| c3896_g1_i1 | related to nonribosomal peptide synthetase MxcG [ IMI 58289] | 2.9 | c8814_g1_i1 | catalase | -2.6 |
| c11811_g1_i1 | putative replication licensing factor | 2.9 | c18486_g1_i1 | pyruvate decarboxylase | -2.6 |
| c15007_g1_i1 | putative CDC7-protein kinase | 2.9 | c13854_g1_i1 | Mx2 protein (GTPase protein) | -2.6 |
| c149_g1_i1 | Fe-S oxidoreductase | 2.9 | c1234_g1_i2 | putative histidine--tRNA ligase, mitochondrial | -2.6 |
| c15627_g1_i1 | putative VPS4-vacuolar sorting protein | 2.9 | c11279_g1_i1 | Chitin deacetylase 1 | -2.5 |
| c17722_g1_i1 | DUR1,2-Urea amidolyase | 2.9 | c1941_g1_i1 | Cu-binding metallothionein | -2.5 |
| c11145_g1_i1 | putative TPR domain protein | 2.8 | c13983_g1_i1 | cofilin | -2.5 |
| c4579_g1_i1 | putative dioxygenase | 2.8 | c6989_g1_i1 | transcription activator | -2.5 |
| c18426_g1_i1 | peroxidase | 2.8 | c2343_g1_i1 | protoporphyrinogen oxidase | -2.6 |
| c15759_g1_i1 | poly(rC)-binding protein 3 | 2.8 | c15781_g1_i1 | vacuolar membrane protein HMT1 (heavy metal tolerance protein) | -2.6 |
| c13310_g1_i1 | probable isocitrate lyase [ IMI 58289] | 2.8 | c5230_g1_i3 | isoleucine tRNA ligase | -2.6 |
| c17023_g1_i1 | SAM-dependent methyltransferase | 2.8 | c13383_g1_i1 | putative stearoyl-CoA desaturase | -2.6 |
| c17703_g1_i1 | NOP14-nuclear and nucleolar protein with possible role in ribosome biogenesis | 2.8 | c8814_g1_i1 | catalase | -2.6 |
| c8648_g2_i1 | putative chaperonin ClpB | 2.8 | c18486_g1_i1 | pyruvate decarboxylase | -2.6 |
| c15588_g1_i1 | NADH oxidase | 2.8 | c13854_g1_i1 | Mx2 protein (GTPase protein) | -2.6 |
| c4360_g1_i1 | DNA repair protein Nse1 | 2.8 | c1234_g1_i2 | putative histidine--tRNA ligase, mitochondrial | -2.6 |
| c5096_g1_i2 | sna41 protein | 2.8 | c11279_g1_i1 | Chitin deacetylase 1 | -2.5 |
| c1374_g1_i2 | FMN-dependent 2-nitropropane dioxygenase | 2.8 | c1941_g1_i1 | Cu-binding metallothionein | -2.5 |
| c4110_g1_i2 | TGL4-triacylglycerol lipase | 2.8 | c13983_g1_i1 | cofilin | -2.5 |
| c1124_g1_i1 | putative translation elongation factor eEF-3 | 2.8 | c6989_g1_i1 | transcription activator | -2.5 |
| c18862_g1_i1 | cytochrome b-large subunit | 2.8 | c3770_g2_i1 | arginase | -2.5 |
| c4935_g1_i2 | related to GABA transport protein [ IMI 58289] | 2.8 | c1056_g1_i1 | interferon-regulated resistance GTP-binding protein | -2.5 |
| c14226_g1_i1 | lactonohydrolase | 2.8 | c2632_g1_i1 | anthranilate synthase component | -2.5 |
| c2695_g1_i1 | putative GCV3-glycine decarboxylase, subunit H | 2.8 | c15580_g1_i1 | putative low-affinity hexose transporter HXT3 | -2.5 |
| c9135_g1_i1 | probable CDC21-thymidylate synthase [ IMI 58289] | 2.8 | c5411_g1_i2 | putative oligosaccharyltransferase alpha subunit | -2.5 |
| c18086_g1_i1 | ATP-dependent RNA helicase ROK1 | 2.8 | c8613_g1_i1 | dnase1 protein | -2.5 |
| c13536_g1_i1 | signal recognition particle protein | 2.8 | c6364_g4_i1 | probable manganese transport protein [ IMI 58289] | -2.5 |
| c8444_g1_i1 | putative pyruvate formate lyase activating enzyme | 2.8 | c6005_g1_i1 | putative hydroxyacylglutathione hydrolase | -2.5 |
| c2661_g1_i2 | 1-phosphatidylinositol-4,5-bisphosphate phosphodiesterase | 2.8 | c1139_g1_i1 | RSB1-integral membrane transporter | -2.5 |
| c5101_g1_i1 | putative YVH1-protein tyrosine phosphatase | 2.8 | c19584_g1_i1 | 4-carboxymuconolactone decarboxylase family protein | -2.5 |
| c5283_g1_i3 | KP4 killer toxin | 2.8 | c835_g1_i1 | beta-galactosidase | -2.5 |
| c1924_g2_i1 | related to cytosine C5-DNA methyltransferase [IMI 58289] | 2.7 | c4344_g1_i1 | putative BRT1 protein, down-regulated by mating factor B | -2.5 |
| c17906_g1_i1 | putative transmembrane protein | 2.7 | c5875_g1_i4 | mRNA splicing factor | -2.5 |
| c6478_g1_i1 | putative CCT6-component of chaperonin-containing T-complex (zeta subunit) | 2.7 | c5219_g1_i1 | multicopper oxidase | -2.5 |
| c16340_g1_i1 | putative DEAD box protein (putative RNA helicase) | 2.7 | c8957_g1_i1 | glutamic acid decarboxylase | -2.5 |
| c18050_g1_i1 | putative translation initiation factor eIF-2 gamma chain | 2.7 | c14051_g1_i1 | putative porphobilinogen synthase | -2.4 |
| c3425_g1_i2 | putative G2/mitotic-specific cyclin B | 2.7 | c5870_g1_i3 | putative LYS7-copper chaperone for superoxide dismutase Sod1p | -2.4 |
| c646_g1_i1 | putative DNA-directed RNA polymerase III second-largest chain | 2.7 | c344_g1_i2 | DUF124 domain protein | -2.4 |
| c5208_g1_i2 | probable heat shock protein 70 [ IMI 58289] | 2.7 | c7937_g1_i1 | alcohol oxidase | -2.4 |
| c18413_g1_i1 | putative replication factor C 38K chain | 2.7 | c2155_g2_i1 | uvs2 protein | -2.4 |
| c88_g1_i1 | meiotically up-regulated protein | 2.7 | c16342_g1_i1 | alpha-1,3-mannosyltransferase | -2.4 |
| c18353_g1_i1 | putative replication licensing factor MCM4 | 2.7 | c18085_g1_i1 | putative aspartate-semialdehyde dehydrogenase | -2.4 |
| c13384_g1_i1 | putative tryptophan--tRNA ligase | 2.7 | c5219_g1_i2 | related to multicopper oxidase [ IMI 58289] | -2.4 |
| c8878_g1_i1 | putative GFA1-glucosamine--fructose-6-phosphate transaminase | 2.7 | c9474_g1_i1 | putative acetyl-CoA synthetase | -2.4 |
| c15566_g1_i1 | TMA16 Protein putative involved in cytoplasmic ribosome function | 2.7 | c7279_g1_i1 | dienelactone hydrolase s | -2.4 |
| c1571_g2_i1 | putative autophagy protein (Atg22) | 2.7 | c14601_g1_i1 | 2-dehydropantoate 2-reductase | -2.4 |
| c4121_g1_i2 | cell wall glycosyl hydrolase YteR | 2.7 | c3183_g1_i1 | polyketide synthase [ IMI 58289] | -2.4 |
| c2495_g1_i1 | kinetoplast-associated protein KAP | 2.7 | c5172_g1_i2 | protein TOL | -2.4 |
| c17700_g1_i1 | STU1-mitotic spindle protein | 2.7 | c3231_g1_i2 | glutathione S-transferase | -2.4 |
| c2018_g1_i2 | high affinity methionine permease | 2.7 | c14565_g1_i1 | putative alpha-L-fucosidase precursor | -2.4 |
| c7433_g1_i1 | putative ISD11 Iron-Sulfur protein biogenesis, Desulfurase-interacting protein | 2.7 | c4306_g1_i2 | putative potassium transporter TRK-1 | -2.4 |
| c15677_g1_i1 | tRNA dihydrouridine synthase | 2.7 | c5848_g1_i4 | putative RPE1-D-ribulose-5-phosphate 3-epimerase | -2.4 |
| c15624_g1_i1 | putative THS1-threonyl tRNA synthetase, cytosolic | 2.7 | c3422_g1_i2 | putative galactose oxidase precursor | -2.4 |
| c3622_g1_i1 | VanZ domain protein | 2.7 | c5473_g1_i1 | malic acid transport protein | -2.4 |
| c16133_g1_i1 | putative COP9 signalosome subunit 5 CSN5 | 2.7 | c8668_g1_i1 | 5-carboxyvanillate decarboxylase | -2.4 |
| c13617_g1_i1 | UPF0183 domain protein | 2.7 | c15622_g1_i1 | DHA14-like major facilitator efflux transporter | -2.3 |
| c3717_g1_i1 | putative spi1-GTP-binding protein | 2.7 | c11469_g1_i1 | Ester hydrolase C11orf54 | -2.3 |
| c6459_g1_i1 | putative small subunit of ribonucleotide reductase | 2.7 | c2621_g1_i2 | TFIID and SAGA subunit TAF61 | -2.3 |
| c8479_g1_i1 | verA protein | 2.7 | c8898_g1_i1 | TPN1 Pyridoxine transporter | -2.3 |
| c17845_g1_i1 | probable GTP-binding protein [ IMI 58289] | 2.7 | c19861_g1_i1 | tol protein | -2.3 |
| c7473_g1_i1 | putative pectate lyase 1 | 2.7 | c9183_g1_i1 | putative DUF895 domain membrane protein | -2.3 |
| c8746_g1_i1 | manganese resistance protein | 2.7 | c6429_g1_i1 | SNF5-component of SWI/SNF transcription activator complex | -2.3 |
| c5871_g1_i3 | UTP-ammonia ligase, partial | 2.7 | c9263_g1_i1 | aspartic proteinase, pepstatin-sensitive | -2.3 |
| c5495_g1_i2 | protein-tyrosine-phosphatase | 2.7 | c1992_g1_i1 | galactose oxidase precursor | -2.3 |
| c6679_g1_i1 | nuclear distribution protein RO11 | 2.7 | c4763_g1_i2 | nucleotide exsicion repair protein RAD7 | -2.3 |
| c13617_g1_i1 | UPF0183 domain protein | 2.7 | c6030_g1_i2 | putative DNA repair protein | -2.3 |
| c8622_g1_i1 | putative proliferating cell nuclear antigen | 2.7 | c4983_g1_i3 | sterigmatocystin 7-O-methyltransferase precursor | -2.3 |
| c1484_g1_i1 | putative isopentenyl-diphosphate delta-isomerase | 2.7 | c18141_g1_i1 | n-alkane-inducible cytochrome P450 | -2.3 |
| c11039_g1_i1 | DBF4-regulatory subunit for Cdc7p protein kinase | 2.7 | c8992_g1_i1 | TRK1-Potassium transporter I | -2.3 |
| c6127_g1_i5 | dna polymerase delta small subunit | 2.6 | c2076_g1_i1 | putative coproporphyrinogen oxidase precursor | -2.3 |
| c6495_g1_i1 | putative nucleolar protein 10 | 2.6 | c2367_g1_i1 | hard surface induced protein 3 (chip3) | -2.3 |
| c9109_g1_i1 | putative APE2-aminopeptidase yscII | 2.6 | c5982_g1_i4 | NADPH-dependent aldehyde reductase | -2.3 |
| c5303_g1_i1 | putative actin-related protein RO7 | 2.6 | c13613_g1_i1 | pisatin demethylase (cytochrome P450) | -2.3 |
| c11471_g1_i1 | succinate-semialdehyde dehydrogenase | 2.6 | c9769_g1_i1 | putative TfdA family oxidoreductase | -2.3 |
| c9816_g1_i1 | cholinesterase | 2.6 | c144_g1_i1 | putative proline racemase | -2.3 |
| c4307_g1_i1 | RPC34-DNA-directed RNA polymerase III | 2.6 | c16563_g1_i1 | related to vegetatible incompatibility protein HET-E-1 [ IMI 58289] | -2.3 |
| c6906_g1_i1 | putative isovaleryl-CoA dehydrogenase | 2.6 | c18102_g1_i1 | cytosolic Cu/Zn superoxide dismutase | -2.3 |
| c329_g1_i1 | putative amidophosphoribosyltransferase | 2.6 | c11877_g1_i1 | related to putative glutathione S-transferase [ IMI 58289] | -2.3 |
| c8840_g1_i1 | putative SCF complex member Cullin 1 | 2.6 | c18884_g1_i1 | heterokaryon incompatibility protein | -2.3 |
| c3351_g1_i1 | Sorbitol utilization protein SOU1 | 2.6 | c4933_g1_i1 | two-component response regulator | -2.3 |
| c6020_g1_i3 | putative chromosome segregation protein cut14 | 2.6 | c4043_g1_i1 | ELG1 protein required for S phase progression and telomere homeostasis | -2.3 |
| c15944_g1_i1 | putative ribosomal protein S25.e.c7 | 2.6 | c11207_g1_i1 | Vault poly | -2.2 |
| c11525_g1_i1 | COP9 signalosome complex subunit 3 | 2.6 | c1768_g1_i1 | related to putative C2H2 zinc finger protein flbC [ IMI 58289] | -2.2 |
| c17669_g1_i1 | response regulator Mcs4 | 2.6 | c5982_g1_i2 | NADPH-dependent aldehyde reductase | -2.2 |
| c2241_g1_i1 | importin beta-2 subunit (transportin) | 2.6 | c5972_g1_i2 | TAD2-tRNA-specific adenosine deaminase 2 | -2.2 |
| c3156_g1_i1 | transcription factor TFIIH chain p47 | 2.6 | c2247_g1_i2 | putative heterogeneous nuclear ribonucleoprotein HRP1 | -2.2 |
| c13620_g1_i1 | thioredoxin-like protein | 2.6 | c5976_g1_i3 | tryptophan dimethylallyltransferase | -2.2 |
| c79_g1_i1 | putative alpha-glucuronidase precursor | 2.6 | c10707_g1_i1 | PAF acetylhydrolase family protein | -2.2 |
| c16042_g1_i1 | putative nucleolar protein | 2.6 | c6659_g1_i1 | phosphatidylserine decarboxylase | -2.2 |
| c4938_g2_i1 | myosin heavy chain | 2.6 | c9859_g1_i1 | lipase 1 | -2.2 |
| c5124_g1_i2 | Transcriptional activator of protease prtT | 2.6 | c2318_g1_i1 | nuclear envelope protein NEM1 | -2.2 |
| c13787_g1_i1 | metaphase-anaphase transition protein (Mlo2) | 2.6 | c6254_g1_i4 | malate dehydrogenase (oxaloacetate-decarboxylating) (NADP+) | -2.2 |
| c17748_g1_i1 | putative APC1-subunit of anaphase-promoting complex (cyclosome) | 2.6 | c13555_g1_i1 | transcription factor atf1+ | -2.2 |
| c6739_g1_i1 | HD family hydrolase | 2.6 | c13342_g1_i1 | mixed-linked glucanase precursor MLG1 | -2.2 |
| c4132_g1_i2 | 26S proteasome-associated ubiquitin carboxyl-terminal hydrolase | 2.6 | c3577_g1_i1 | cytochrome c oxidase subunit 1 (mitochondrion) | -2.2 |
| c4287_g1_i1 | replication factor C protein | 2.6 | c9695_g1_i1 | transaminase type I | -2.2 |
| c2241_g1_i2 | importin beta-2 subunit (transportin) | 2.6 | c8731_g1_i1 | SUR2 protein | -2.2 |
| c4996_g2_i1 | putative CPR6-member of the cyclophilin family | 2.6 | c3824_g1_i1 | putative alkaline protease (oryzin) | -2.2 |
| c2976_g1_i1 | putative translation initiation factor eIF-4A | 2.6 | c4196_g1_i1 | beta transducin-like protein | -2.2 |
| c6691_g1_i1 | rho coiled-coil associated kinase alpha | 2.6 | c11871_g1_i1 | NonF protein, involved in nonactin biosynthesis | -2.2 |
| c13833_g1_i1 | putative AVT3-involved in amino acid efflux from the vacuole | 2.6 | c11098_g1_i1 | soluble fumarate reductase (NADH) | -2.2 |
| c8665_g1_i1 | transcriptional activator CMR1 | 2.6 | c9614_g1_i1 | related to tetracycline resistance proteins [ IMI 58289] | -2.2 |
| c10501_g1_i1 | DNA damage-responsive protein 48 | 2.6 | c13421_g1_i1 | large-conductance mechanosensitive channel | -2.2 |
| c7108_g1_i1 | RNA helicase | 2.6 | c5804_g1_i2 | laccase precursor | -2.2 |
| c17814_g1_i1 | alpha-glucosidase b | 2.6 | c9887_g1_i1 | serine-type carboxypeptidase Z precursor | -2.2 |
| c18543_g1_i1 | nicotinate phosphoribosyltransferase | 2.6 | c8981_g1_i1 | vacuolar protein sorting VACUOLELESS1 | -2.2 |
| c13541_g1_i1 | putative RNA helicase involved in ribosome biogenesis | 2.6 | c6372_g2_i7 | short chain dehydrogenase | -2.2 |
| c4877_g1_i1 | ribonuclease P protein subunit p29 | 2.6 | c6378_g1_i16 | bifunctional GTP cyclohydrolase II/3, 4-dihydroxy-2butanone-4-phosphate synthase | -2.2 |
| c15659_g1_i1 | putative malate dehydrogenase | 2.6 | c4055_g1_i2 | related to helicase-like transcription factor protein [ IMI 58289] | -2.2 |
| c6130_g2_i1 | Mac1p interacting protein (MIC1 protein) | 2.6 | c5273_g1_i3 | SRC1-regulation of cohesion (Splice variant I) | -2.2 |
| c3894_g1_i1 | cullulin 3 | 2.6 | c3839_g1_i1 | increased rDNA silencing protein IRS4 | -2.2 |
| c18568_g1_i1 | Pwp2p | 2.5 | c3801_g1_i1 | putative fructose-2,6-bisphosphate 2-phosphatase | -2.2 |
| c18214_g1_i1 | putative U5 snRNP-specific protein | 2.5 | c11599_g1_i1 | GAL4-like transcriptional activator | -2.2 |
| c3541_g1_i2 | pseudouridine synthase | 2.5 | c2504_g1_i1 | related to esterase [ IMI 58289] | -2.2 |
| c12103_g1_i1 | phenol hydroxylase | 2.5 | c1967_g2_i1 | related to GAL4-like transcriptional activator [ IMI 58289] | -2.2 |
| c517_g1_i1 | carboxylesterase type B | 2.5 | c9727_g1_i1 | GNT1 alphaN-acetylglucosamine transferase | -2.2 |
| c17872_g1_i1 | translation initiation factor eIF3 | 2.5 | c18223_g1_i1 | major facilitator (MFS1) transporter | -2.2 |
| c5941_g1_i2 | ADH4-alcohol dehydrogenase IV | 2.5 | c18365_g1_i1 | aminopeptidase | -2.2 |
| c18589_g1_i1 | dithiol-disulfide isomerase involved in polyketide biosynthesis | 2.5 | c3929_g1_i3 | related to DUF159 domain protein [ IMI 58289] | -2.2 |
| c9357_g1_i1 | proliferation associated SNF2-like protein | 2.5 | c10631_g1_i1 | beta-mannosidase | -2.2 |
| c460_g1_i1 | putative homogentisate 1,2-dioxygenase | 2.5 | c5042_g1_i1 | glomerulosclerosis protein Mpv17 | -2.1 |
| c5286_g1_i1 | putative ATP-binding multidrug cassette transport protein | 2.5 | c11892_g1_i1 | NmrA-like family protein | -2.1 |
| c5278_g1_i1 | putative NMT1-N-myristoyltransferase | 2.5 | c4319_g1_i4 | protein N-acetyltransferase NAT2 | -2.1 |
| c8835_g1_i1 | putative CCT7-component of chaperonin-containing T-complex | 2.5 | c2817_g1_i3 | GIT1-Glycerophosphoinositol transporter also able to mediate low-affinity phosphate trans | -2.1 |
| c1908_g1_i1 | putative methionine synthase, vitamin-b12 independent | 2.5 | c2670_g1_i2 | mannosylphosphorylation protein MNN4 | -2.1 |
| c10958_g1_i1 | signal recognition particle protein Sec65 | 2.5 | c3799_g1_i1 | putative catalytic subunit of DNA polymerase zeta UPR-1 | -2.1 |
| c16803_g1_i1 | exosome complex exonuclease | 2.5 | c2719_g1_i2 | glutathione transferase omega 1 | -2.1 |
| c18899_g1_i1 | reductase | 2.5 | c11065_g1_i1 | MFS multidrug transporter | -2.1 |
| c3711_g1_i1 | hypothetical protein LW93_8367 | 2.5 | c4231_g1_i2 | integral membrane protein, Mpv17/PMP22 family | -2.1 |
| c6974_g1_i1 | putative serine/threonine protein kinase COT-1 | 2.5 | c18201_g1_i1 | protein MCH2 (monocarboxylate permease) | -2.1 |
| c6882_g1_i1 | putative tailless complex polypeptide 1 / chaperonin subunit alpha | 2.5 | c1867_g1_i1 | BCS1 protein | -2.1 |
| c1833_g1_i1 | formamidopyrimidine-DNA glycosylase | 2.5 | c13325_g2_i1 | maleylacetate reductase | -2.1 |
| c18143_g1_i1 | putative protein kinase ck2 catalytic subunit ck2 alpha-3 | 2.5 | c516_g1_i1 | putative beta-succinyl CoA synthetase precursor | -2.1 |
| c11780_g1_i1 | aldose 1-epimerase | 2.5 | c3009_g1_i1 | thermoresistant gluconokinase | -2.1 |
| c3162_g1_i1 | micromolar calcium activated neutral protease 1 (capn1) | 2.5 | c8881_g1_i1 | SUR1-required for mannosylation | -2.1 |
| c3706_g2_i1 | SDA1 protein, required for normal organization of the actin cytoskeleton | 2.5 | c2207_g1_i1 | putative nicotinate-nucleotide pyrophosphorylase (carboxylating) | -2.1 |
| c5095_g1_i2 | mannosyltransferase | 2.5 | c5236_g1_i3 | related to meiotically up-regulated gene 72 protein [ IMI 58289] | -2.1 |
| c4746_g2_i1 | zinc/cadmium resistance protein | 2.5 | c6321_g1_i5 | RNA binding protein Nrd1 | -2.1 |
| c13518_g1_i1 | putative bifunctional purine biosynthetic protein | 2.5 | c6106_g1_i2 | related to monocarboxylate transporter 2 [ IMI 58289] | -2.1 |
| c5783_g1_i1 | zinc transporter | 2.5 | c2903_g1_i1 | putative holocytochrome-c synthase | -2.1 |
| c17672_g2_i1 | probable DNA mismatch repair protein MSH2 [ IMI 58289] | 2.5 | c2799_g1_i1 | srpA precursor | -2.1 |
| c1972_g1_i1 | microtubule associated protein | 2.5 | c4420_g1_i1 | related to AAH1-adenosine deaminase [ IMI 58289] | -2.1 |
| c6416_g1_i1 | putative ABC1 transport protein | 2.5 | c6104_g1_i3 | protein-lysine N-methyltransferase | -2.1 |
| c11823_g1_i1 | putative 2-haloalkanoic acid dehalogenase | 2.5 | c4443_g1_i1 | putative phosphate transport protein, mitochondrial | -2.1 |
| c9205_g1_i1 | putative dityrosine transporter | 2.5 | c11369_g1_i1 | cyclin CCL1 | -2.1 |
| c9520_g1_i1 | bifunctional 4-hydroxyphenylacetate degradation enzyme | 2.5 | c4016_g1_i1 | putative synaptobrevin (v-SNARE) | -2.1 |
| c6992_g1_i1 | putative phenylacetyl-CoA ligase | 2.5 | c55_g1_i2 | transcription factor RGM1 | -2.1 |
| c5018_g1_i2 | protein kinase CDC5 | 2.5 | c6315_g1_i3 | putative inosine triphosphate pyrophosphatase | -2.1 |
| c11440_g1_i1 | zinc finger protein | 2.5 | c4052_g1_i2 | putative threonine aldolase | -2.1 |
| c13322_g1_i1 | MET10-sulfite reductase flavin-binding subunit | 2.5 | c6055_g1_i3 | nuclear localization protein NPL6 | -2.1 |
| c4599_g1_i1 | PHO87 protein | 2.5 | c13330_g1_i1 | PNG1-protein with de-N-glycosylation function (N-glycanase) | -2.1 |
| c210_g1_i1 | putative ribonucleoside-diphosphate reductase large chain (un-24) | 2.5 | c7368_g1_i1 | multidrug resistant protein | -2.1 |
| c9649_g1_i1 | KM-PA-2 protein | 2.5 | c14063_g1_i1 | phosphatidylinositol phospholipase | -2.1 |
| c14297_g1_i1 | phenylacetaldoxime dehydratase family protein | 2.5 | c7096_g1_i1 | cAMP-dependent protein kinase catalytic subunit | -2.1 |
| c3919_g2_i1 | G alpha subunit | 2.5 | c2060_g1_i1 | endocytosis ankyrin repeat protein Nuc-2 | -2.1 |
| c4312_g1_i1 | putative ribosomal elongation factor EF-2 | 2.5 | c7234_g1_i1 | O-methyltransferase | -2.1 |
| c5849_g1_i3 | probable leucine--tRNA ligase, cytosolic [ IMI 58289] | 2.5 | c2191_g1_i2 | DNA mismatch repair protein PMS2 | -2.1 |
| c6523_g1_i1 | component of the anaphase promoting complex | 2.5 | c6772_g1_i1 | ferric-chelate reductase | -2.1 |
| c3523_g2_i1 | probable NDE1-mitochondrial cytosolically directed NADH dehydrogenase [ IMI 58289] | 2.5 | c7901_g1_i1 | carbonic anhydrase | -2.1 |
| c3341_g1_i1 | probable c-14 sterol reductase ERG-3 [ IMI 58289] | 2.5 | c17885_g1_i1 | alcohol dehydrogenase Bli-4 | -2.1 |
| c2602_g1_i1 | DNA-directed RNA polymerase III chain RPC82 | 2.5 | c17444_g1_i1 | trp-asp repeat containing protein | -2.1 |
| c3714_g2_i1 | L-fucose dehydrogenase | 2.5 | c6253_g1_i1 | senescence marker protein 30 | -2.1 |
| c3649_g1_i1 | NAM7-nonsense-mediated mRNA decay protein | 2.5 | c12013_g1_i1 | vegetatible incompatibility protein HET-E-1 | -2.1 |
| c5344_g1_i1 | putative RNA helicase DBP5 | 2.5 | c1053_g1_i1 | choline monooxygenase | -2.0 |
| c14062_g1_i1 | lysophospholipase L1 and related esterase | 2.5 | c2553_g1_i1 | putative protein involved in intramitochondrial protein sorting | -2.0 |
| c4872_g1_i1 | rabkinesin-6 | 2.5 | c5645_g1_i1 | translocation protein | -2.0 |
| c14655_g1_i1 | protein kinase | 2.5 | c3417_g1_i1 | putative Alcohol dehydrogenase | -2.0 |
| c18248_g1_i1 | putative ketol-acid reductoisomerase (ilv-2) | 2.5 | c4055_g1_i1 | helicase-like transcription factor protein | -2.0 |
| c5284_g1_i1 | probable sulfite reductase (NADPH) [ IMI 58289] | 2.5 | c4488_g1_i2 | probable thiol-specific antioxidant [ IMI 58289] | -2.0 |
| c5739_g1_i2 | putative arginyl-tRNA synthetase, cytosolic | 2.4 | c1718_g1_i1 | ADH7-NADP(H)-dependent alcohol dehydrogenase | -2.0 |
| c13481_g1_i1 | putative glycogen synthase | 2.4 | c928_g1_i1 | putative Protein required for hyphal anastomosis (HAM-2) | -2.0 |
| c647_g1_i2 | amidohydrolase AmhX | 2.4 | c6372_g3_i2 | high-affinity phosphate permease, phosphate-repressible | -2.0 |
| c6844_g1_i1 | putative FAP7-involved in the oxidative stress response | 2.4 | c2348_g2_i1 | TOB3 (member of AAA-ATPase family) | -2.0 |
| c11435_g1_i1 | DnaJ subfamily A member 2 | 2.4 | c4291_g1_i1 | phytase | -2.0 |
| c15832_g1_i1 | putative fluconazole resistance protein | 2.4 | c7676_g1_i1 | putative 3-demethylubiquinone-9 3-methyltransferase | -2.0 |
| c15743_g1_i1 | putative COP9 complex subunit 2 | 2.4 | c2428_g1_i1 | putative transcription factor Pig1p | -2.0 |
| c1846_g1_i1 | embryogenesis abundant protein | 2.4 | c2019_g1_i1 | related to fumonisin cluster-transcription factor [ IMI 58289] | -2.0 |
| c1130_g1_i2 | UTP22-U3 snoRNP protein involved in maturation of pre-18S rRNA | 2.4 | c6312_g1_i4 | PBN1 protein, required for post-translational processing of the protease B precursor Prb1p | -2.0 |
| c6449_g1_i1 | putative EB1-like protein | 2.4 | c19548_g1_i1 | putative conidiation protein con-6 | -2.0 |
| c13879_g1_i1 | art-4 protein | 2.4 | c5640_g1_i4 | acetate regulatory DNA binding protein FacB | -2.0 |
| c14208_g1_i1 | Lactam utilization protein lamB | 2.4 | c18390_g1_i1 | gibberellin 20-oxidase | -2.0 |
| c15687_g1_i1 | putative ATP-dependent RNA helicase | 2.4 | c5125_g1_i2 | isoflavone reductase | -2.0 |
| c5428_g1_i2 | beta-carotene 15,15`-dioxygenase | 2.4 |  |  |  |
| c11167_g1_i1 | acyl-CoA thioester hydrolase, mitochondrial precursor | 2.4 |  |  |  |
| c6163_g1_i4 | putative protein kinase DBF20 | 2.4 |  |  |  |
| c9103_g1_i1 | putative FPR3-prolyl cis-trans isomerase | 2.4 |  |  |  |
| c16077_g1_i1 | GTP-binding protein beta subunit-like protein | 2.4 |  |  |  |
| c17728_g3_i2 | putative GTPase Rho | 2.4 |  |  |  |
| c17868_g1_i1 | dehydrogenase s | 2.4 |  |  |  |
| c3540_g1_i1 | regulator of Ty1 transposition | 2.4 |  |  |  |
| c18514_g1_i1 | putative DIP2-Dom34p-interacting protein | 2.4 |  |  |  |
| c11600_g1_i1 | HOL1, putative substrate-H+ antiporter | 2.4 |  |  |  |
| c17682_g1_i1 | mitochondrial intermediate peptidase precursor | 2.4 |  |  |  |
| c13603_g1_i1 | Transketolase | 2.4 |  |  |  |
| c5055_g2_i2 | transcriptional repressor rco-1 | 2.4 |  |  |  |
| c6532_g1_i1 | rhodopsin kinase | 2.4 |  |  |  |
| c11228_g1_i1 | quinate transport protein | 2.4 |  |  |  |
| c13337_g1_i1 | raptor, a binding partner of target of rapamycin (TOR) | 2.4 |  |  |  |
| c1382_g1_i1 | delta3-cis-delta2-trans-enoyl-CoA isomerase | 2.4 |  |  |  |
| c6729_g1_i1 | putative ran GTPase activating protein 1 | 2.4 |  |  |  |
| c3528_g1_i1 | structure-specific recognition protein | 2.4 |  |  |  |
| c16903_g1_i1 | fumarate reductase flavoprotein subunit precursor | 2.4 |  |  |  |
| c5170_g1_i1 | MRD1 protein | 2.4 |  |  |  |
| c2637_g1_i1 | putative rcd1 protein involved in sexual development | 2.4 |  |  |  |
| c18384_g1_i1 | ubiquitin-specific protease 7 | 2.4 |  |  |  |
| c18228_g1_i1 | SKI3-antiviral protein | 2.4 |  |  |  |
| c938_g1_i2 | putative acetylxylan esterase | 2.4 |  |  |  |
| c16653_g1_i1 | multidrug resistant protein | 2.4 |  |  |  |
| c8754_g1_i1 | putative branched-chain alpha-keto acid dehydrogenase complex | 2.4 |  |  |  |
| c14769_g1_i1 | ARCA protein | 2.4 |  |  |  |
| c2558_g2_i1 | aminotriazole resistance protein | 2.4 |  |  |  |
| c2840_g1_i1 | putative monosaccharide transporter | 2.4 |  |  |  |
| c5060_g1_i2 | phosphoglycerate mutase | 2.4 |  |  |  |
| c11160_g1_i1 | importin beta | 2.4 |  |  |  |
| c8807_g1_i1 | putative importin alpha SRP1 | 2.4 |  |  |  |
| c18407_g1_i1 | putative translation initiation factor 3 (eIF-3) zeta subunit | 2.4 |  |  |  |
| c3467_g1_i1 | putative HYP2-translation initiation factor eIF5A.1 | 2.4 |  |  |  |
| c17688_g1_i1 | RNA binding protein | 2.4 |  |  |  |
| c1631_g1_i1 | ubiquitin--protein ligase | 2.4 |  |  |  |
| c4192_g1_i1 | carrier protein YMC1, mitochondrial | 2.4 |  |  |  |
| c5644_g1_i1 | related to C2H2 zinc finger protein [ IMI 58289] | 2.4 |  |  |  |
| c795_g1_i1 | putative peroxisomal amine oxidase | 2.4 |  |  |  |
| c18524_g1_i1 | aerobactin siderophore biosynthesis protein iucB | 2.4 |  |  |  |
| c3773_g1_i1 | Phosphoserine phosphatase | 2.4 |  |  |  |
| c8876_g1_i1 | related to myo-inositol transport protein ITR1 [ IMI 58289] | 2.4 |  |  |  |
| c2635_g1_i2 | putative isoleucine--tRNA ligase | 2.4 |  |  |  |
| c19195_g1_i1 | DAL81-transcriptional activator | 2.4 |  |  |  |
| c13245_g1_i1 | triacylglycerol lipase V precursor | 2.4 |  |  |  |
| c1164_g1_i1 | PIF1 protein precursor | 2.4 |  |  |  |
| c8742_g1_i1 | putative catalase 2 | 2.4 |  |  |  |
| c4292_g1_i1 | D-arabinitol 2-dehydrogenase | 2.4 |  |  |  |
| c4990_g1_i1 | programmed cell death protein (calcium-binding protein) | 2.3 |  |  |  |
| c4407_g1_i2 | chromate transport protein | 2.3 |  |  |  |
| c15952_g1_i1 | hydroxymethylglutaryl-CoA lyase | 2.3 |  |  |  |
| c13488_g1_i1 | related to membrane protein [ IMI 58289] | 2.3 |  |  |  |
| c5191_g1_i3 | DUF255 domain protein | 2.3 |  |  |  |
| c1050_g1_i2 | positive activator of transcription | 2.3 |  |  |  |
| c16164_g1_i1 | PUF6 Member of the PUF protein family | 2.3 |  |  |  |
| c357_g1_i2 | putative osmotic sensitive-2 protein (putative mitogen-activated protein (MAP) kinase) | 2.3 |  |  |  |
| c16068_g1_i1 | putative TSR1 Protein | 2.3 |  |  |  |
| c10988_g1_i1 | putative sugar transporter | 2.3 |  |  |  |
| c5063_g1_i1 | benzoate 4-monooxygenase cytochrome P450 | 2.3 |  |  |  |
| c2949_g1_i1 | putative purine nucleoside permease | 2.3 |  |  |  |
| c6933_g1_i1 | probable glucan 1,3-beta-glucosidase [ IMI 58289] | 2.3 |  |  |  |
| c5561_g1_i5 | transcription factor Pig1p | 2.3 |  |  |  |
| c5852_g1_i2 | queuine-tRNA ribosyltransferase | 2.3 |  |  |  |
| c2275_g1_i1 | rRNA-processing protein UTP23 | 2.3 |  |  |  |
| c3538_g1_i1 | putative RNase L inhibitor | 2.3 |  |  |  |
| c5092_g1_i1 | phosphoprotein phosphatase 2C | 2.3 |  |  |  |
| c5871_g1_i1 | CTP synthase | 2.3 |  |  |  |
| c9293_g1_i1 | GLY1-L-threonine aldolase, low-specific | 2.3 |  |  |  |
| c17909_g1_i1 | putative Pre-mRNA-splicing factor CWC21 | 2.3 |  |  |  |
| c12144_g1_i1 | positive transcriptional regulator for purine utilization | 2.3 |  |  |  |
| c6283_g1_i1 | tubulin-folding cofactor D (chaperone) | 2.3 |  |  |  |
| c6144_g1_i1 | thiol methyltransferase | 2.3 |  |  |  |
| c6942_g1_i1 | ubiquitin fusion degradation protein | 2.3 |  |  |  |
| c5849_g1_i1 | probable leucine--tRNA ligase, cytosolic [ IMI 58289] | 2.3 |  |  |  |
| c11607_g1_i1 | pectinesterase | 2.3 |  |  |  |
| c8818_g1_i1 | putative glutathione reductase (NADPH) | 2.3 |  |  |  |
| c2395_g2_i1 | maltose permease | 2.3 |  |  |  |
| c13543_g1_i1 | putative Chitin Synthase 1 (chs-1) | 2.3 |  |  |  |
| c6897_g1_i1 | CIC1 Adaptor protein | 2.3 |  |  |  |
| c6474_g1_i1 | DAHP synthase class II | 2.3 |  |  |  |
| c5376_g1_i2 | putative SH3-domain protein Cyk3 | 2.3 |  |  |  |
| c5571_g2_i1 | general repressor of transcription | 2.3 |  |  |  |
| c2118_g1_i1 | TMA46-Protein putative involved in cytoplasmic ribosome function | 2.3 |  |  |  |
| c10507_g1_i1 | peptide transporter | 2.3 |  |  |  |
| c4225_g1_i1 | transmembrane transporter Liz1p | 2.3 |  |  |  |
| c13538_g1_i1 | putative Hsp90 co-chaperone Cdc37 | 2.3 |  |  |  |
| c1307_g1_i2 | putative CDS1-CDP-diacylglycerol synthase | 2.3 |  |  |  |
| c18261_g1_i1 | dihydrodipicolinate synthase | 2.3 |  |  |  |
| c6159_g2_i1 | related to Vacuolar membrane-associated protein iml-1 [ IMI 58289] | 2.3 |  |  |  |
| c17826_g1_i1 | adaptin ear-binding coat-associated protein 1 | 2.3 |  |  |  |
| c15606_g1_i1 | putative nucleolar protein NOP58 | 2.3 |  |  |  |
| c4579_g1_i3 | putative dioxygenase | 2.3 |  |  |  |
| c14000_g1_i1 | growth regulation protein WHI2 | 2.3 |  |  |  |
| c1052_g1_i1 | fungal transcriptional regulatory protein | 2.3 |  |  |  |
| c4337_g2_i1 | putative sepB protein | 2.3 |  |  |  |
| c17775_g1_i1 | pyrroline-5-carboxylate reductase | 2.3 |  |  |  |
| c4496_g1_i3 | vesicular amine transporter | 2.3 |  |  |  |
| c1029_g1_i1 | peptidylprolyl isomerase (cyclophilin)-like protein | 2.3 |  |  |  |
| c4263_g1_i2 | LSM1 protein | 2.3 |  |  |  |
| c2667_g1_i2 | putative choline-sulfatase | 2.3 |  |  |  |
| c192_g1_i2 | 2,5-diketo-D-gluconic acid reductase | 2.3 |  |  |  |
| c2050_g1_i2 | putative DNA replication regulator SLD2 | 2.3 |  |  |  |
| c6993_g1_i1 | aflatoxin efflux pump AFLT | 2.3 |  |  |  |
| c2440_g1_i2 | putative cpc-3 protein | 2.3 |  |  |  |
| c8938_g1_i1 | purine nucleotide binding protein | 2.3 |  |  |  |
| c2327_g1_i1 | transcription activator protein acu-15 | 2.3 |  |  |  |
| c18562_g1_i1 | cleft lip and palate transmembrane protein 1 (CLPTM1) | 2.3 |  |  |  |
| c17856_g1_i1 | probable fibrillarin (NOP1) [ IMI 58289] | 2.3 |  |  |  |
| c4149_g1_i2 | chloride-bicarbonate anion exchanger AE2 | 2.3 |  |  |  |
| c9581_g1_i1 | GTP-binding protein | 2.3 |  |  |  |
| c786_g1_i1 | cold sensitive U2 snRNA supressor | 2.3 |  |  |  |
| c4816_g1_i1 | probable SUP35-eukaryotic peptide chain release factor GTP-binding subunit [ IMI 58289] | 2.3 |  |  |  |
| c4132_g1_i3 | 26S proteasome-associated ubiquitin carboxyl-terminal hydrolase | 2.3 |  |  |  |
| c16005_g1_i1 | putative methylcrotonoyl-CoA carboxylase biotin carboxylase chain | 2.3 |  |  |  |
| c11072_g1_i1 | TOK1-voltage-gated, outward-rectifying K+ channel | 2.3 |  |  |  |
| c7141_g1_i1 | probable aldehyde dehydrogenase [ IMI 58289] | 2.3 |  |  |  |
| c13279_g1_i1 | putative transketolase | 2.3 |  |  |  |
| c3354_g1_i1 | severin kinase | 2.2 |  |  |  |
| c2505_g1_i2 | endopeptidase La 2 | 2.2 |  |  |  |
| c18234_g1_i1 | antigen LSA-1 | 2.2 |  |  |  |
| c6941_g1_i1 | putative beta transducin-like protein | 2.2 |  |  |  |
| c6285_g1_i1 | URK1 protein | 2.2 |  |  |  |
| c4245_g1_i1 | putative potassium channel beta subunit protein | 2.2 |  |  |  |
| c13748_g1_i1 | transcriptional regulator RDS2 | 2.2 |  |  |  |
| c6482_g1_i1 | putative ribosomal protein L14.e.B, cytosolic | 2.2 |  |  |  |
| c6901_g1_i1 | putative t-complex-type molecular chaperone, epsilon subunit | 2.2 |  |  |  |
| c3039_g1_i1 | importin9 | 2.2 |  |  |  |
| c4635_g1_i1 | POL12 (DNA-directed DNA polymerase alpha) | 2.2 |  |  |  |
| c6029_g1_i3 | UDP-N-acetylglucosamine-peptide N-acetylglucosaminyltransferase | 2.2 |  |  |  |
| c7181_g1_i1 | putative isp4 protein | 2.2 |  |  |  |
| c3330_g1_i1 | metallo-beta-lactamase family protein | 2.2 |  |  |  |
| c5404_g2_i2 | putative DNA polymerase epsilon, calytic chain POL2 | 2.2 |  |  |  |
| c7550_g1_i1 | putative isoamyl alcohol oxidase | 2.2 |  |  |  |
| c4679_g1_i2 | heterokaryon incompatibility protein (het-6OR allele) | 2.2 |  |  |  |
| c5530_g1_i1 | component of actin cortical patches LAS17 | 2.2 |  |  |  |
| c5711_g1_i1 | ABC1 transport protein | 2.2 |  |  |  |
| c5654_g1_i1 | beta transducin-like protein | 2.2 |  |  |  |
| c3085_g1_i1 | probable cutinase transcription factor 1 beta [ IMI 58289] | 2.2 |  |  |  |
| c6584_g1_i1 | beta-tubulin | 2.2 |  |  |  |
| c803_g1_i1 | metalloproteinase | 2.2 |  |  |  |
| c4774_g1_i1 | salicylate 1-monooxygenase (flavoprotein monooxygenase) | 2.2 |  |  |  |
| c13508_g1_i1 | putative zuotin | 2.2 |  |  |  |
| c4589_g1_i1 | putative RIB2-DRAP deaminase | 2.2 |  |  |  |
| c4186_g1_i1 | phosphotyrosyl phosphatase activator PTPA | 2.2 |  |  |  |
| c5989_g1_i1 | small nuclear ribonucleoprotein U2B | 2.2 |  |  |  |
| c3980_g1_i2 | putative ketopantoate reductase family protein | 2.2 |  |  |  |
| c2339_g1_i2 | cop9 (constitutive photomorphogenic), subunit 6 | 2.2 |  |  |  |
| c5148_g1_i1 | cytochrome P450 | 2.2 |  |  |  |
| c4878_g1_i1 | ATP dependent RNA helicase | 2.2 |  |  |  |
| c3945_g1_i1 | putative recombinational repair protein mus-23 | 2.2 |  |  |  |
| c5486_g1_i2 | putative nuclear protein localization factor and ER translocation component NPL4 | 2.2 |  |  |  |
| c5428_g1_i1 | beta-carotene 15,15`-dioxygenase | 2.2 |  |  |  |
| c4027_g1_i1 | prefoldin subunit 3 | 2.2 |  |  |  |
| c8695_g1_i1 | aspartate--tRNA ligase | 2.2 |  |  |  |
| c4946_g1_i1 | Ras GTPase-activating-like protein IQGAP1 | 2.2 |  |  |  |
| c827_g1_i2 | TBP-binding repressor protein | 2.2 |  |  |  |
| c11463_g1_i1 | RING finger domain protein | 2.2 |  |  |  |
| c2153_g1_i1 | putative profilin | 2.2 |  |  |  |
| c16305_g1_i1 | putative PTR2-Di-and tripeptide permease | 2.2 |  |  |  |
| c3647_g1_i1 | ATPase | 2.2 |  |  |  |
| c6190_g1_i2 | chloride channel protein 3 | 2.2 |  |  |  |
| c3431_g1_i1 | cornifin B | 2.2 |  |  |  |
| c9545_g1_i1 | putative NOC4 nucleolar protein | 2.2 |  |  |  |
| c15890_g1_i1 | UTP10-U3 snoRNP protein | 2.2 |  |  |  |
| c5961_g1_i1 | nuclear protein SA-1 | 2.2 |  |  |  |
| c701_g1_i1 | nuclear pore membrane protein | 2.2 |  |  |  |
| c5599_g1_i2 | SRP40-suppressor of mutant AC40 of RNA polymerase I and III | 2.2 |  |  |  |
| c9661_g1_i1 | putative glucosamine-phosphate N-acetyltransferase | 2.2 |  |  |  |
| c6365_g4_i2 | 3-oxoacyl-[acyl-carrier-protein] reductase | 2.2 |  |  |  |
| c6741_g1_i1 | 5-methylcytosine G/T mismatch-specific DNA glycosylase | 2.2 |  |  |  |
| c4059_g1_i1 | cell wall mannoprotein | 2.2 |  |  |  |
| c3102_g1_i1 | putative oxidoreductase CipA-like | 2.2 |  |  |  |
| c17742_g1_i1 | DNA topoisomerase II | 2.2 |  |  |  |
| c3562_g1_i1 | CYC8-general repressor of transcription | 2.2 |  |  |  |
| c4398_g1_i1 | COP9 signalosome subunit 4 | 2.2 |  |  |  |
| c6750_g1_i1 | ubiquitin-conjugating enzyme | 2.2 |  |  |  |
| c4032_g2_i1 | elongation factor G | 2.2 |  |  |  |
| c8954_g1_i1 | putative carbamoyl-phosphate synthase (glutamine-hydrolyzing) arginine-specific large chain | 2.2 |  |  |  |
| c13717_g1_i1 | spindle assembly checkpoint protein | 2.2 |  |  |  |
| c4412_g1_i2 | fluconazole resistance protein (FLU1) | 2.2 |  |  |  |
| c6629_g1_i1 | putative Golgi apparatus membrane protein TVP23 | 2.2 |  |  |  |
| c8617_g1_i1 | putative mitochondrial receptor complex chain MOM22 | 2.2 |  |  |  |
| c6415_g1_i1 | putative adenosylhomocysteinase (S-adenosyl-L-homocysteine hydrolase) | 2.2 |  |  |  |
| c4481_g1_i2 | related to WD-repeat protein CRB3 [ IMI 58289] | 2.2 |  |  |  |
| c11577_g1_i1 | CCR4 protein | 2.2 |  |  |  |
| c2339_g1_i1 | related to sorbitol dehydrogenase [ IMI 58289] | 2.2 |  |  |  |
| c4486_g1_i2 | dna-binding protein | 2.2 |  |  |  |
| c4780_g1_i1 | putative 1-phosphatidylinositol 3-kinase | 2.2 |  |  |  |
| c6484_g1_i1 | putative ribosomal protein L12 | 2.2 |  |  |  |
| c913_g1_i1 | translation initiation factor GCD7 | 2.2 |  |  |  |
| c18272_g1_i1 | putative flavin-containing monooxygenase | 2.2 |  |  |  |
| c4980_g2_i1 | ribosomal RNA processing protein RRP5 | 2.2 |  |  |  |
| c6481_g1_i1 | putative PAB1-mRNA polyadenylate-binding protein | 2.2 |  |  |  |
| c17930_g1_i1 | pre-mRNA splicing factor U2AF large chain | 2.2 |  |  |  |
| c2004_g1_i2 | putative LST8 protein | 2.2 |  |  |  |
| c8690_g1_i1 | USO1-intracellular protein transport protein | 2.2 |  |  |  |
| c3957_g1_i1 | kinase | 2.2 |  |  |  |
| c788_g1_i1 | multidrug resistant protein | 2.2 |  |  |  |
| c3976_g1_i2 | histone methyltransferase | 2.2 |  |  |  |
| c9100_g1_i1 | putative ferrochelatase | 2.2 |  |  |  |
| c8393_g1_i1 | gibberellin biosynthesis-related | 2.2 |  |  |  |
| c4521_g1_i2 | C6 finger domain protein | 2.2 |  |  |  |
| c16471_g1_i1 | tyrosinase precursor (monophenol monooxygenase) | 2.1 |  |  |  |
| c6557_g1_i1 | putative mitotic control protein dis3+ | 2.1 |  |  |  |
| c5579_g1_i3 | putative URA2-multifunctional pyrimidine biosynthesis protein | 2.1 |  |  |  |
| c13429_g1_i1 | malonyl CoA synthetase | 2.1 |  |  |  |
| c3056_g1_i1 | putative L-arabinitol 4-dehydrogenase | 2.1 |  |  |  |
| c13591_g1_i1 | Survival factor 1 | 2.1 |  |  |  |
| c5789_g1_i1 | transport protein USO1 | 2.1 |  |  |  |
| c3864_g2_i1 | UPC2-regulatory protein involved in control of sterol uptake | 2.1 |  |  |  |
| c386_g2_i1 | related to xylosidase/arabinosidase [ IMI 58289] | 2.1 |  |  |  |
| c5841_g1_i3 | putative Histone-lysine N-methyltransferase SET9 | 2.1 |  |  |  |
| c13664_g1_i1 | putative argininosuccinate lyase | 2.1 |  |  |  |
| c16544_g1_i1 | putative CAF4 protein | 2.1 |  |  |  |
| c996_g2_i1 | putative UVSB PI-3 kinase | 2.1 |  |  |  |
| c18027_g1_i1 | probable RNA helicase, mitochondrial [ IMI 58289] | 2.1 |  |  |  |
| c18245_g1_i1 | arabinose 5-phosphate isomerase | 2.1 |  |  |  |
| c5035_g2_i1 | putative SWR1-DEAH-box protein, putative RNA helicase | 2.1 |  |  |  |
| c5302_g1_i4 | dityrosine transporter | 2.1 |  |  |  |
| c2656_g2_i1 | probable RPP1A-60S large subunit acidic ribosomal protein a1 [ IMI 58289] | 2.1 |  |  |  |
| c1346_g1_i1 | putative phosphatidylglycerophosphate synthase PEL1 | 2.1 |  |  |  |
| c4598_g1_i3 | ferroxidase | 2.1 |  |  |  |
| c687_g1_i1 | lipase/esterase | 2.1 |  |  |  |
| c16737_g1_i1 | related to ADA regulatory protein of adaptive response [ IMI 58289] | 2.1 |  |  |  |
| c19558_g1_i1 | putative ATP-binding protein PRP16 | 2.1 |  |  |  |
| c207_g1_i1 | CUE3 Meiotic induced protein | 2.1 |  |  |  |
| c11404_g1_i1 | phytanoyl-CoA dioxygenase family protein | 2.1 |  |  |  |
| c73_g1_i1 | ATP-dependent DNA helicase II | 2.1 |  |  |  |
| c3538_g1_i2 | U1 snRNP protein | 2.1 |  |  |  |
| c18625_g1_i1 | flavin-containing monooxygenase | 2.1 |  |  |  |
| c13798_g1_i1 | MAP kinase | 2.1 |  |  |  |
| c16773_g1_i1 | mitochondrial Fe2+ transporter, MMT1 family | 2.1 |  |  |  |
| c6497_g1_i1 | related to regulatory protein alcR [ IMI 58289] | 2.1 |  |  |  |
| c3464_g1_i1 | Rho guanyl nucleotide exchange factor | 2.1 |  |  |  |
| c1156_g1_i2 | putative heterokaryon incompatibility Het-C protein | 2.1 |  |  |  |
| c17877_g1_i1 | antheridiol steroid receptor | 2.1 |  |  |  |
| c17991_g1_i1 | tuftelin-interacting protein 11 | 2.1 |  |  |  |
| c7502_g1_i1 | putative D-amino-acid oxidase | 2.1 |  |  |  |
| c3820_g1_i1 | putative translation initiation factor eIF-2 alpha chain | 2.1 |  |  |  |
| c17116_g1_i1 | GPI anchored protein | 2.1 |  |  |  |
| c1952_g1_i1 | related to killer toxin insensitive protein 3 [ IMI 58289] | 2.1 |  |  |  |
| c17914_g1_i1 | major facilitator MirA | 2.1 |  |  |  |
| c10031_g1_i1 | ARO80-positive transcription regulator of ARO9 and ARO10 | 2.1 |  |  |  |
| c5750_g1_i3 | putative Na+-transporting ATPase ENA-1 | 2.1 |  |  |  |
| c15767_g1_i1 | cycloheximide-inducible protein CIP70 (cytochrome P450 family) | 2.1 |  |  |  |
| c5928_g1_i2 | geranylgeranyl transferase alpha chain | 2.1 |  |  |  |
| c16024_g1_i1 | nucleoporin-interacting protein NIC96 | 2.1 |  |  |  |
| c9687_g1_i1 | negative regulator of transcription from Pol II promoter | 2.1 |  |  |  |
| c237_g1_i1 | putative IDP1-isocitrate dehydrogenase (NADP+), mitochondrial | 2.1 |  |  |  |
| c11738_g1_i1 | putative methyltransferase DPH5 | 2.1 |  |  |  |
| c5732_g1_i1 | replication protein CDC23 | 2.1 |  |  |  |
| c5951_g1_i3 | long-chain fatty-acid-CoA ligase | 2.1 |  |  |  |
| c16762_g1_i1 | nonribosomal peptide synthetase | 2.1 |  |  |  |
| c4786_g1_i2 | related to nitrate assimilation regulatory protein nirA [ IMI 58289] | 2.1 |  |  |  |
| c6364_g7_i1 | cytochrome P450 7B1 | 2.1 |  |  |  |
| c5207_g1_i3 | putative isocitrate dehydrogenase (NAD) | 2.1 |  |  |  |
| c1962_g1_i1 | myotubularin related protein 1 | 2.1 |  |  |  |
| c6469_g1_i1 | hypothetical protein Y057_6115 | 2.1 |  |  |  |
| c4124_g1_i3 | monocarboxylate transporter 4 | 2.1 |  |  |  |
| c2142_g1_i2 | carbohydrate kinase, contains PfkB domain | 2.1 |  |  |  |
| c6222_g1_i2 | inosine-uridine preferring nucleoside hydrolase | 2.1 |  |  |  |
| c5803_g1_i1 | putative D-xylulose kinase | 2.1 |  |  |  |
| c13331_g1_i1 | putative heat-shock protein hsp60 | 2.1 |  |  |  |
| c16750_g1_i1 | nicotinamide mononucleotide permease | 2.1 |  |  |  |
| c15608_g1_i1 | putative valine--tRNA ligase | 2.1 |  |  |  |
| c6454_g1_i1 | putative SIK1-involved in pre-rRNA processing | 2.1 |  |  |  |
| c1149_g1_i1 | putative pre-mRNA splicing factor prp1 | 2.1 |  |  |  |
| c8653_g1_i1 | putative ornithine aminotransferase | 2.1 |  |  |  |
| c8341_g1_i1 | putative norsolorinic acid reductase | 2.1 |  |  |  |
| c11086_g1_i1 | general amidase | 2.1 |  |  |  |
| c4723_g1_i2 | related to heterokaryon incompatibility protein (het-6OR allele) [ IMI 58289] | 2.1 |  |  |  |
| c6157_g1_i1 | related to alpha-mannosidase [ IMI 58289] | 2.1 |  |  |  |
| c10157_g1_i1 | sexual differentiation process protein | 2.1 |  |  |  |
| c6156_g1_i2 | N-acetylglucosaminyl-phosphatidylinositol biosynthetic protein gpi1 | 2.1 |  |  |  |
| c5082_g1_i3 | putative ubiquitin-protein ligase UBC4 | 2.1 |  |  |  |
| c2210_g1_i1 | putative GTP-binding protein rac1 | 2.1 |  |  |  |
| c1582_g1_i2 | sporulation protein SPO72 | 2.1 |  |  |  |
| c2583_g1_i2 | putative HAT2-subunit of the major histone acetyltransferase | 2.1 |  |  |  |
| c11802_g1_i1 | ribonuclease H1 | 2.1 |  |  |  |
| c1558_g1_i1 | putative serine-tRNA ligase, cytosolic | 2.1 |  |  |  |
| c16121_g1_i1 | putative UTP7-U3 snoRNP protein | 2.1 |  |  |  |
| c4401_g1_i2 | GCD6-translation initiation factor eIF2b epsilon | 2.1 |  |  |  |
| c9177_g1_i1 | putative GLO1-glyoxalase I | 2.1 |  |  |  |
| c7161_g1_i1 | related to 5`-3` DNA helicase [ IMI 58289] | 2.1 |  |  |  |
| c3597_g1_i2 | peroxisomal targeting signal receptor | 2.1 |  |  |  |
| c17866_g1_i1 | putative brefeldin A resistance protein | 2.1 |  |  |  |
| c14554_g1_i1 | putative DUR1,2-urea amidolyase | 2.1 |  |  |  |
| c4147_g2_i1 | polycomb group protein MEDEA | 2.1 |  |  |  |
| c2640_g2_i1 | putative class I alpha-mannosidase | 2.1 |  |  |  |
| c4216_g1_i3 | putative calcium/proton exchanger | 2.1 |  |  |  |
| c1429_g1_i1 | putative methionine adenosyltransferase ETH-1 | 2.1 |  |  |  |
| c3971_g1_i1 | DUF803 domain membrane protein | 2.1 |  |  |  |
| c13217_g1_i1 | peptide transport protein | 2.1 |  |  |  |
| c14161_g1_i1 | related to TRANSKETOLASE [ IMI 58289] | 2.1 |  |  |  |
| c15865_g1_i1 | putative tryptophan synthase | 2.1 |  |  |  |
| c13460_g1_i1 | related to BENZOYLFORMATE DECARBOXYLASE [ IMI 58289] | 2.1 |  |  |  |
| c6188_g1_i4 | multidrug resistance-associated protein | 2.1 |  |  |  |
| c15631_g1_i1 | putative lysine-tRNA ligase | 2.1 |  |  |  |
| c8897_g1_i1 | condensin complex component cnd2 | 2.1 |  |  |  |
| c1240_g1_i2 | large neutral amino acid transporter | 2.1 |  |  |  |
| c5778_g1_i4 | BUD20 Protein involved in bud-site selection | 2.1 |  |  |  |
| c12281_g1_i1 | phosphotidylinositol kinase | 2.1 |  |  |  |
| c4146_g1_i2 | putative ATPase component of chromatin remodeling complex (ISW1) | 2.1 |  |  |  |
| c191_g1_i1 | SIP2-dominant suppressor of some ts mutations in RPO21 and PRP4 | 2.1 |  |  |  |
| c4747_g1_i1 | putative arrestin domain protein | 2.1 |  |  |  |
| c7458_g1_i1 | putative ribosomal protein L26 | 2.1 |  |  |  |
| c17750_g1_i1 | cullin 4A | 2.1 |  |  |  |
| c11162_g1_i1 | putative 3-hydroxy-3-methylglutaryl-coenzyme A reductase | 2.1 |  |  |  |
| c4403_g1_i3 | related to C6 transcription factor [ IMI 58289] | 2.1 |  |  |  |
| c15238_g1_i1 | alkaline protease (oryzin) | 2.1 |  |  |  |
| c2099_g1_i2 | sensory transduction histidine kinase | 2.1 |  |  |  |
| c9225_g1_i1 | Ctr copper transporter | 2.1 |  |  |  |
| c18501_g1_i1 | nuclear assembly factor NAF1 | 2.1 |  |  |  |
| c674_g1_i1 | putative translation elongation factor EF-Tu precursor, mitochondrial | 2.1 |  |  |  |
| c118_g1_i1 | VPS62-Vacuolar Protein Sorting | 2.0 |  |  |  |
| c17789_g1_i1 | putative proline-tRNA ligase | 2.0 |  |  |  |
| c6682_g1_i1 | putative 40s ribosomal protein s11.e, cytosolic | 2.0 |  |  |  |
| c1278_g1_i1 | ATP-binding-cassette protein | 2.0 |  |  |  |
| c2350_g1_i2 | spindle pole body protein | 2.0 |  |  |  |
| c5323_g1_i1 | putative GTP-binding protein | 2.0 |  |  |  |
| c11251_g1_i1 | putative polyketide synthase | 2.0 |  |  |  |
| c328_g1_i2 | putative glucokinase | 2.0 |  |  |  |
| c8841_g1_i1 | putative RPL34B-ribosomal protein L34.e | 2.0 |  |  |  |
| c6220_g1_i2 | DUF895 domain membrane protein | 2.0 |  |  |  |
| c7066_g1_i1 | monophenol monooxygenase (tyrosinase) | 2.0 |  |  |  |
| c19123_g1_i1 | related to pisatin demethylase [ IMI 58289] | 2.0 |  |  |  |
| c19516_g1_i1 | related to ankyrin 1 [ IMI 58289] | 2.0 |  |  |  |
| c5256_g1_i2 | mitochondrial rRNA processing protein PRP12 | 2.0 |  |  |  |
| c5814_g1_i1 | ankyrin 3 | 2.0 |  |  |  |
| c2724_g1_i1 | putative ATP dependent RNA helicase | 2.0 |  |  |  |
| c3964_g1_i2 | related to Ras guanine-nucleotide exchange protein Cdc25p [ IMI 58289] | 2.0 |  |  |  |
| c1979_g1_i1 | cellulose binding protein CEL1 | 2.0 |  |  |  |
| c6148_g2_i1 | budding protein | 2.0 |  |  |  |
| c9212_g1_i1 | probable cytosolic nonspecific dipeptidase [ IMI 58289] | 2.0 |  |  |  |
| c5914_g1_i1 | putative mitotic checkpoint serine/threonine protein kinase | 2.0 |  |  |  |
| c13256_g1_i1 | putative S3 ribosomal protein | 2.0 |  |  |  |
| c6208_g1_i2 | methyl transferase | 2.0 |  |  |  |
| c18708_g1_i1 | lipase/acylhydrolase | 2.0 |  |  |  |
| c1660_g1_i1 | putative DNA repair protein MUS-42 | 2.0 |  |  |  |
| c2468_g1_i1 | zinc-binding protein | 2.0 |  |  |  |
| c6661_g1_i1 | related to spore coat protein SP96 precursor [ IMI 58289] | 2.0 |  |  |  |
| c4836_g1_i1 | putative glycine hydroxymethyltransferase, cytosolic | 2.0 |  |  |  |
| c19392_g1_i1 | mfs-multidrug-resistance transporter | 2.0 |  |  |  |
| c4929_g1_i3 | putative MOB1 protein | 2.0 |  |  |  |
| c11134_g1_i1 | putative alpha-tubulin B | 2.0 |  |  |  |
| c14097_g1_i1 | UBX5-UBX (ubiquitin regulatory X) domain-containing protein | 2.0 |  |  |  |
| c6001_g1_i3 | related to chitinase [ IMI 58289] | 2.0 |  |  |  |
| c16659_g1_i1 | Cupin domain protein | 2.0 |  |  |  |
| c7015_g1_i1 | F-box protein PpaB | 2.0 |  |  |  |
| c7114_g1_i1 | midasin (AAA ATPase) | 2.0 |  |  |  |
| c6663_g1_i1 | putative METHIONYL-TRNA SYNTHETASE, mitochondrial | 2.0 |  |  |  |
| c6970_g1_i1 | kinesin-like protein kif1c | 2.0 |  |  |  |
| c3595_g1_i2 | (AAP1)-alanine/arginine aminopeptidase | 2.0 |  |  |  |
| c6196_g1_i1 | CSN12-signalosome component | 2.0 |  |  |  |
| c9054_g1_i1 | endoplasmic reticulum transmembrane protein | 2.0 |  |  |  |
| c2574_g1_i2 | pig tubulin-tyrosine ligase | 2.0 |  |  |  |
| c15629_g1_i1 | putative ubiquitin-specific processing protease 21 | 2.0 |  |  |  |
| c6397_g1_i1 | endo-1,4-beta-xylanase | 2.0 |  |  |  |
| c15479_g1_i1 | DUF1680 domain protein | 2.0 |  |  |  |
| c5959_g1_i2 | bifunctional polynucleotide phosphatase/kinase | 2.0 |  |  |  |
| c4830_g1_i3 | translation elongation factor HBS1 protein | 2.0 |  |  |  |
| c11707_g1_i1 | cell division control protein CDC18+ | 2.0 |  |  |  |
| c14251_g1_i1 | enoyl-CoA hydratase precursor, mitochondrial | 2.0 |  |  |  |
| c3924_g1_i1 | putative deoxyribodipyrimidine photo-lyase PHR | 2.0 |  |  |  |
| c13576_g1_i1 | putative RPL33B-ribosomal protein L35a.e.c15 | 2.0 |  |  |  |
| c16328_g1_i1 | amino acid permease 2 (AAP-2) | 2.0 |  |  |  |
| c4295_g1_i1 | Fre1p and Fre2p | 2.0 |  |  |  |
| c6069_g1_i1 | ral2 protein | 2.0 |  |  |  |
| c13974_g1_i1 | phospholipase a-2-activating protein | 2.0 |  |  |  |
| c5114_g1_i2 | putative Arf-binding protein | 2.0 |  |  |  |
| c4939_g1_i1 | polymyositis-scleroderma protein | 2.0 |  |  |  |
| c6094_g1_i1 | putative MNN10-subunit of mannosyltransferase complex | 2.0 |  |  |  |
| c5139_g1_i3 | Deoxyhypusine hydroxylase | 2.0 |  |  |  |
| c18194_g1_i1 | putative uracil-DNA glycosylase | 2.0 |  |  |  |
| c6425_g1_i1 | transporter protein | 2.0 |  |  |  |
| c6368_g1_i4 | RTA1-involved in 7-aminocholesterol resistance | 2.0 |  |  |  |
| c4005_g2_i1 | hexose transporter protein | 2.0 |  |  |  |
| c18156_g1_i1 | putative glycine--tRNA ligase GRS1 | 2.0 |  |  |  |
| c3986_g1_i1 | putative nucleosome assembly protein I | 2.0 |  |  |  |
| c8929_g1_i1 | H2A histone H2A | 2.0 |  |  |  |
| c4809_g1_i1 | decapping enzyme | 2.0 |  |  |  |
| c16230_g1_i1 | related to histidine kinase tcsA protein [ IMI 58289] | 2.0 |  |  |  |
| c5907_g1_i3 | bifunctional P-protein (chorismate mutase-P/prephenate dehydratase) | 2.0 |  |  |  |
| c10970_g1_i1 | putative acyl-CoA dehydrogenase | 2.0 |  |  |  |
| c6010_g1_i2 | putative calcium P-type ATPase NCA-3 (Ca2+-transporting ATPase) | 2.0 |  |  |  |
| c17717_g1_i1 | 3`-5` exonuclease | 2.0 |  |  |  |
| c19132_g1_i1 | pathway-specific regulatory protein nit-4 | 2.0 |  |  |  |
| c17934_g1_i1 | O-methyltransferase B | 2.0 |  |  |  |
| c5929_g1_i4 | calpain-like protease palBory | 2.0 |  |  |  |
| c6657_g1_i1 | putative RCO3 glucose transporter | 2.0 |  |  |  |
| c10995_g1_i1 | acetyl-hydrolase | 2.0 |  |  |  |
| c8692_g1_i1 | ADH5-alcohol dehydrogenase V | 2.0 |  |  |  |
| c3967_g1_i1 | putative calcium P-type ATPase NCA-1 | 2.0 |  |  |  |
| c5355_g1_i1 | cyclin B3 | 2.0 |  |  |  |
| c11839_g1_i1 | putative ascus development protein 3 | 2.0 |  |  |  |
| c18491_g1_i1 | Ser/Thr protein phosphatase superfamily | 2.0 |  |  |  |
| c11051_g1_i1 | putative SED1-abundant cell surface glycoprotein | 2.0 |  |  |  |
| c2624_g1_i1 | COMPASS component SWD1 | 2.0 |  |  |  |
| c4778_g2_i2 | vacuolar sorting protein | 2.0 |  |  |  |
| c5010_g1_i2 | carnitine/acylcarnitine translocase | 2.0 |  |  |  |
| c1702_g1_i2 | putative ketopantoate hydroxymethyltransferase | 2.0 |  |  |  |
| c6209_g1_i3 | SYG1 protein [Fusarium fujikuroi] | 2.0 |  |  |  |
|  |  |  |  |  |  |
